# NuSAP Safeguards Centriole Integrity to Mediate CEP57‐CEP152 Torus Recruitment for Proper Engagement

**DOI:** 10.1002/advs.202515192

**Published:** 2026-01-30

**Authors:** Shiyu Zhang, Zemin Jiang, Qiaoyun Yang, Hong Zheng, Minghao Wang, Chennianci Zhu, Lih‐Wen Deng, Karen Carmelina Crasta, Yih‐Cherng Liou

**Affiliations:** ^1^ Department of Biological Sciences Faculty of Science National University of Singapore Singapore Singapore; ^2^ Department of Thoracic Surgery Xinqiao Hospital Army Medical University Chongqing China; ^3^ Department of Breast and Thyroid Surgery Southwest Hospital Army Medical University Chongqing China; ^4^ Integrative Sciences and Engineering Programme NUS Graduate School National University of Singapore Singapore Singapore; ^5^ Department of Biochemistry Yong Loo Lin School of Medicine National University of Singapore Singapore Singapore; ^6^ NUS Center for Cancer Research Yong Loo Lin School of Medicine National University of Singapore Singapore Singapore; ^7^ Department of Physiology Yong Loo Lin School of Medicine National University of Singapore Singapore Singapore; ^8^ Healthy Longevity Translational Research Programme Yong Loo Lin School of Medicine National University of Singapore Singapore Singapore

**Keywords:** centriole disengagement, centrosomal proteins, centrosome cycle, CEP57, microtubule‐associated protein, NuSAP

## Abstract

Precise centrosome regulation is crucial for faithful cell division, yet how microtubule‐associated proteins (MAPs) contribute to centrosome integrity remains poorly understood. Here, we uncover a novel localization of NuSAP—a RanGTP‐regulated MAP—on centrioles using super‐resolution microscopy. NuSAP depletion disrupts centriole tubulin architecture, triggering premature centriole disengagement, compromising pericentriolar material (PCM) cohesion, and disrupting the spatial organization of the CEP57‐CEP63‐CEP152 torus assembly. Using TurboID‐based proteomics and biochemical assays, we identify CEP57 as a direct interactor of NuSAP. We further demonstrate that NuSAP is essential for the initial recruitment of the CEP57‐CEP63‐CEP152 complex to the proximal end of procentrioles. Based on these findings, we propose a novel two‐step model of torus complex recruitment, in which NuSAP‐dependent stabilization of centriole tubulin is required for early CEP57 loading. This discovery not only resolves a longstanding gap in our understanding of how MAP‐mediated tubulin stabilization contributes to centrosome organization but also provides a mechanistic framework for the hierarchical recruitment of the centriole torus complex.

## Graphical abstract

This study reveals a novel role for the microtubule stabilizer NuSAP at centrioles. NuSAP depletion destabilizes the centriole's tubulin structure, causing premature disengagement, PCM defects, and mis‐localization of the CEP57‐CEP63‐CEP152 complex. By reinforcing centriole architecture, NuSAP enables early CEP57 loading and initiates a newly proposed two‐step pathway for torus complex assembly, PCM assembly, and organization.

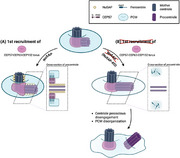

## 1 Introduction

The centrosome serves as the primary microtubule‐organizing center (MTOC) [1, 2] in animal cells, ensuring accurate spindle assembly [3, 4], chromosome segregation [5], and maintenance of genomic stability [6, 7]. Aberrant regulation of centrosome duplication, disjunction, and disengagement during the cell cycle leads to spindle multipolarity [8], chromosome mis‐segregation [9], and aneuploidy [10], which underlie tumorigenesis [11, 12, 13] and developmental disorders such as microcephaly and primordial dwarfism [14, 15, 16, 17].

The centrosome cycle is tightly coupled to centriole duplication [18, 19] and engagement dynamics [20, 21]. Each cell begins G1 with a pair of orthogonally arranged centrioles connected by a proteinaceous linker [22, 23]. During the S phase, procentrioles form adjacent to the mother centrioles, establishing an engaged configuration that prevents reduplication [19, 24]. As cells progress into the G2 phase, centrosomes disjoin through linker dissolution to allow spindle pole separation, while mother–daughter pairs remain engaged [25, 26]. Centriole disengagement occurs at mitotic exit and functions as a licensing step for the next duplication cycle [27, 28]. This process is driven by the coordinated activities of Polo‐like kinase 1 (PLK1) [29, 30, 31, 32] and Separase [33]. Failure to regulate these transitions properly leads to precocious centriole disengagement, which disrupts centrosome function and impairs chromosome segregation [34]. Despite extensive progress, how centriolar structural integrity and engagement are mechanistically coupled remains incompletely understood.

The CEP57–CEP63–CEP152 module forms a toroidal scaffold at the proximal end of centrioles that is essential for centriole engagement, maturation, and duplication licensing [35, 36, 37]. Super‐resolution analyses have revealed a concentric organization of the pericentriolar material (PCM), with CEP57 positioned at the inner layer [37] to recruit CEP63 and CEP152 [38, 39, 40], which together establish the platform for Polo‐like kinase 4 (PLK4) loading [41]. Disruption of CEP57 results in PCM disorganization, centriole disengagement defects, and impaired duplication [42]. Mutations in the *CEP57* gene cause mosaic‐variegated aneuploidy (MVA) syndrome, characterized by chromosomal instability and defective spindle architecture [43, 44, 45, 46]. However, the molecular mechanisms that regulate CEP57 recruitment and assembly of the centriolar torus complex remain unresolved.

Recent research indicates a strong interplay between microtubule assembly and CEP57 recruitment [47], which suggests an interesting nexus between microtubules and microtubule‐associated protein (MAP) with CEP57 and the centriole torus complex. Microtubule‐associated proteins (MAPs) constitute a diverse group of proteins that interact with microtubules, exerting regulatory control over microtubule stability, dynamics, and organization [48, 49, 50]. While their roles in mitosis, cell migration, intracellular transport, and neuronal development have been extensively studied, emerging evidence indicates that a subset of MAPs also contributes to the regulation of centrosome integrity and dynamics [51, 52, 53]. Nucleolar and Spindle‐Associated Protein (NuSAP), encoded by gene *NUSAP1*, is a RanGTP‐regulated MAP that functions as a critical microtubule stabilizer [54]. By bundling and crosslinking microtubules, it ensures the mechanical integrity of the mitotic spindle [54, 55]. Most intriguingly, NuSAP has recently been strongly implicated in overall growth and brain development, as evidenced by two unrelated patients with microcephaly and severe developmental delays who were found to carry the same recurrent *de novo* heterozygous *NUSAP1* mutation [56]. Similarly, mutations in centrosomal genes, including *CENPJ*, *CDK5RAP2*, *CEP63*, and *CEP152*, are also associated with microcephaly and related developmental disorders [16, 17, 57, 58]. Moreover, the expression levels of NuSAP protein, primarily controlled by the anaphase‐promoting complex/cyclosome [59], rise rapidly from the S phase to the G2 phase, coinciding with the critical process of centriole duplication and elongation [60]. Furthermore, it has been reported that NuSAP depletion leads to multipolar spindle formation [60], suggesting a potential role for NuSAP in the regulation of centrosome duplication and engagement during the cell cycle. However, the precise mechanism by which NuSAP maintains centrosome integrity remains largely undefined.

In this study, we discover a novel centriolar function of NuSAP during the G2 phase, demonstrating its pivotal role in preserving centrosomal integrity and coordinating timely centriole pair disengagement. Utilizing advanced microscopic techniques, including Ultrastructure Expansion Microscopy (U‐ExM) and Stimulated Emission Depletion (STED) super‐resolution microscopy, we demonstrate that the depletion of NuSAP leads to precocious centriole disengagement, significant structural abbreviation of PCM, and distortion of centriole tubulin architecture. Furthermore, we show that NuSAP is crucial for the proper recruitment of CEP57 and the CEP57‐CEP63‐CEP152 centriole torus complex to the proximal end of procentrioles. Disruption of the centriole tubulin structure by Vinblastine treatment similarly mimics this mis‐localization, indicating that NuSAP plays a vital role in safeguarding centriole tubulin stability, which is critical for proper recruitment of CEP57 and the centriole torus complex. Finally, we propose a novel two‐step model for CEP57‐CEP63‐CEP152 torus recruitment, in which the first step is NuSAP‐dependent. Overall, this study provides significant insights into the regulatory mechanisms governing centrosome engagement and highlights the versatile role of microtubule‐associated proteins, such as NuSAP, in maintaining the function of cellular organelles.

## 2 Results

### 2.1 Depletion of NuSAP Induces Precocious Centriole Disengagement in the G2 Phase

**FIGURE 1:**
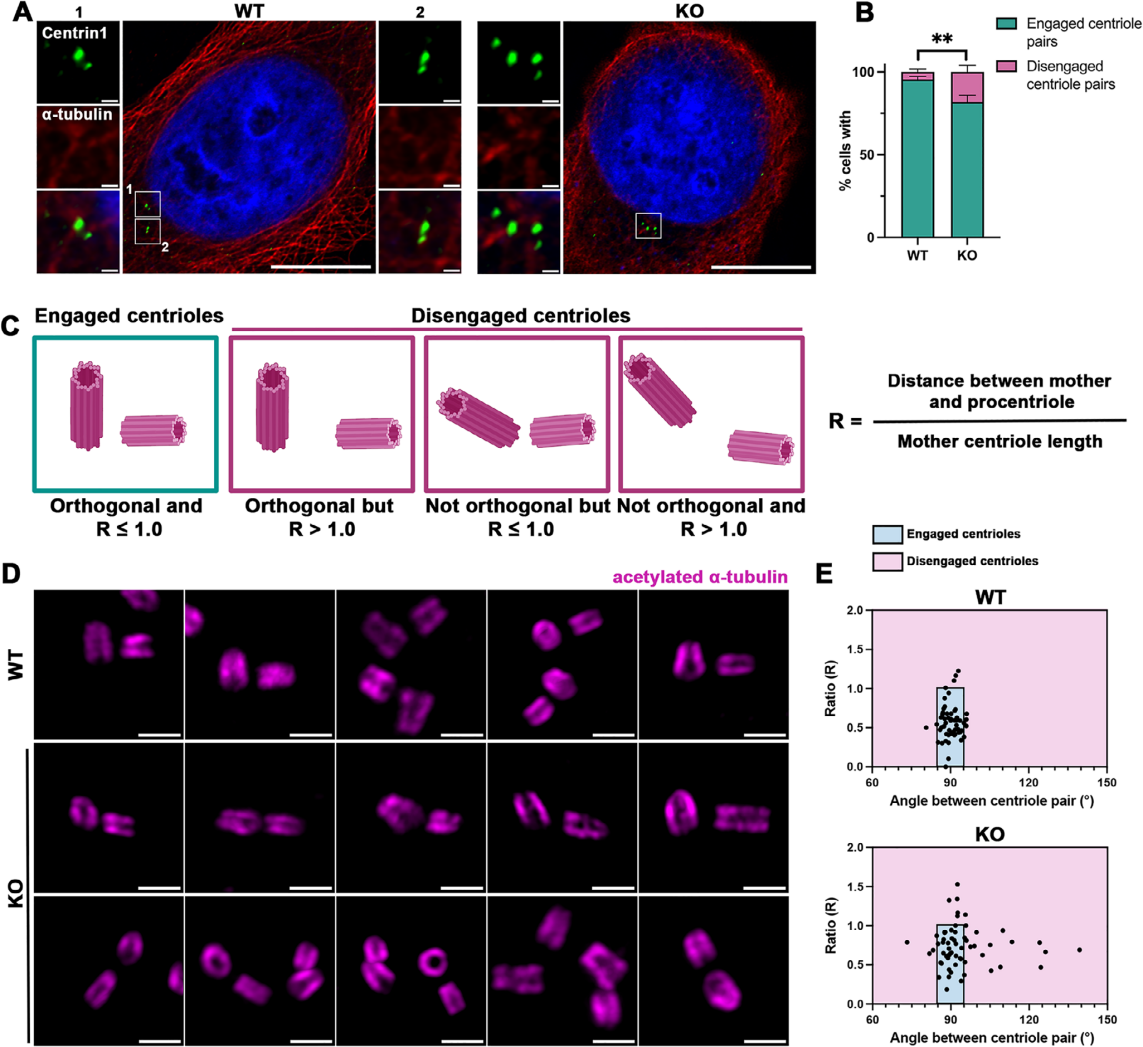
Depletion of NuSAP induces precocious centriole disengagement. (A) WT and NuSAP‐KO HeLa cells were synchronized in the G2 phase and stained for IF with antibodies against α‐tubulin (red) and Centrin1 (green). Scale bar, 10 µm and 0.5 µm in the inserts. (B) Histograms represent the frequency of G2 cells with the dis/engaged centriole pairs in (A). Values are mean percentages ± s.d. from three independent experiments. WT n = 310, KO n = 336. **p < 0.01. (C) Schematic illustrating possible centriole configurations in G2 phase centrosomes and the criteria used to distinguish engaged versus disengaged centriole pairs. (D) U‐ExM analysis of centriole configuration in WT and NuSAP‐KO cells synchronized in the G2 phase. Cells were fixed, expanded according to the U‐ExM protocol, and stained against acetylated α‐tubulin (magenta). (E) Dot plot quantifying centriole orientation based on the ratio of the mother–procentriole distance to mother centriole length and angle between centriole pairs. Each dot represents an individual centriole pair (WT n = 73; NuSAP‐KO n = 67; three independent experiments).

To investigate the potential role of NuSAP in centrosome regulation, we first constructed NuSAP‐knockout HeLa cells (sgRNA, genomic DNA sequencing, and Western blot analysis confirming NuSAP depletion in Figure S1A–C). Given the tight coordination required for centriole engagement and disengagement during the centrosome cycle, we aimed to determine whether NuSAP depletion disrupts centriole integrity during interphase, particularly in the G2 phase, when centriole engagement is crucial for proper mitotic entry. To test this, we synchronized WT and NuSAP‐KO cells in the G2 phase and examined centriole configuration. We observed a significant increase in precociously disengaged centriole pairs in NuSAP‐KO cells in the G2 phase (Figure 1A), with the percentage of cells with precocious centriole pairs increasing from 4.5 ± 1.9% in WT cells to 18.1 ± 4.1% in NuSAP‐KO cells (Figure 1B). Our findings suggest an essential role for NuSAP in centriole engagement to prevent precocious disengagement in the G2 phase.

To assess centriole engagement, we applied the quantitative framework established by Dwivedi et al. [32] and analyzed centriole architecture using ultrastructure expansion microscopy (U‐ExM). Engagement status was determined by measuring the ratio (R) of the inter‐centriole distance to the mother centriole length and the angle between the centriole pair. Centrioles were classified as engaged when R ≤ 1 and the relative angle was 90 ± 5°; pairs failing either criterion were scored as disengaged (schematic in Figure 1C). Using this analysis, U‐ExM imaging revealed a marked increase in disengaged centriole pairs in NuSAP‐KO cells compared with WT (Figure 1D,E). These quantitative measurements are consistent with the conventional immunofluorescence data (Figure 1A) and further support the requirement of NuSAP in proper centriole engagement.

To investigate whether this defect persists beyond the G2 phase, we also examined centriole configuration during mitosis. We found that NuSAP‐KO cells exhibited a higher incidence of multipolar spindle formation (images in Figure S1D, schematic in Figure S1E, and quantification in Figure S1F) and precocious centriole disengagement (images in Figure S1D and quantification in Figure S1G). These findings suggest that the precocious centriole disengagement observed in the G2 phase may contribute to mitotic spindle defects. Our primary focus remains on elucidating how NuSAP regulates centriole integrity in the G2 phase.

### 2.2 NuSAP Depletion Induces Disorganization of PCM in the G2 Phase

**FIGURE 2:**
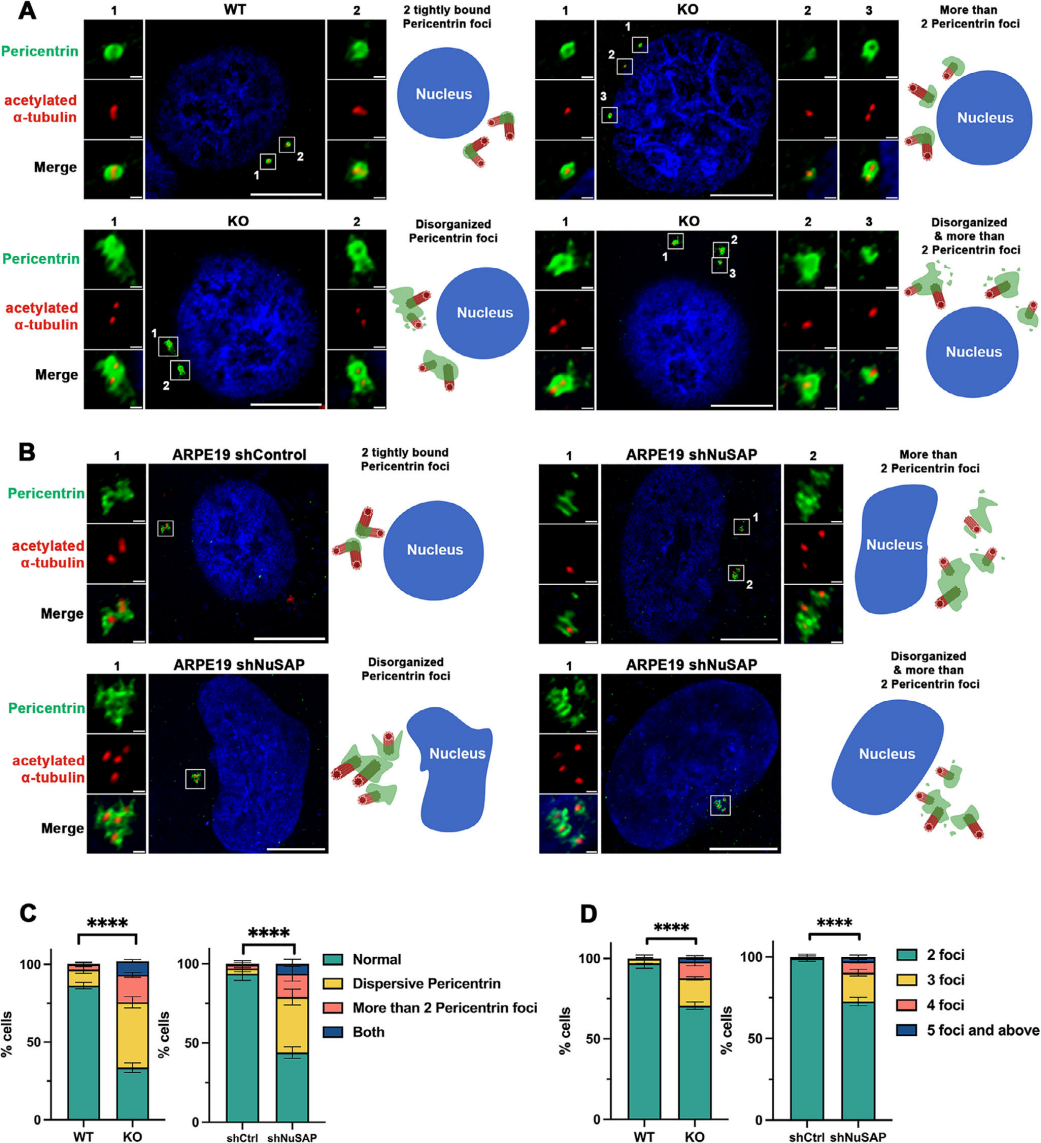
Depletion of NuSAP induces PCM disorganization. (A) WT and NuSAP‐KO HeLa cells were synchronized in the G2 phase and stained for IF with antibodies against acetylated α‐tubulin (red) and pericentrin (green). Scale bar, 10 µm and 0.5 µm in the inserts. Schematic models of the phenotype of NuSAP‐KO observed were illustrated. (B) shControl and shNuSAP in ARPE19 cells were synchronized in the G2 phase and stained for IF with antibodies against acetylated α‐tubulin (red) and pericentrin (green). Scale bar, 10 µm and 0.5 µm in the inserts. Schematic models of the phenotype of NuSAP knockdown observed were illustrated. (C) Histograms represent the frequency of G2 cells with the indicated phenotypes observed in (A) and (B). Values are mean percentages ± s.d. from three independent experiments: WT n = 300, KO n = 300, shControl n = 300, shNuSAP n = 300. (D) Histograms represent the frequency of G2 cells with various pericentrin foci numbers observed in (A) and (B). Values are mean percentages ± s.d. from three independent experiments: WT n = 300, KO n = 300, shControl n = 300, shNuSAP n = 300.

The integrity of the PCM is essential for proper centriole engagement. To determine whether precociously disengaged centrioles in NuSAP‐KO cells exhibit PCM defects, we assessed the organization of pericentrin, a major PCM scaffold protein. During the G2 phase, WT cells displayed the expected PCM architecture, characterized by two tightly bound pericentrin rings surrounding each centriole pair (Figure 2A, upper left). Consistently, 86.3 ± 2.1% of WT cells showed this tightly clustered pericentrin foci (Figure 2C, green bar). In contrast, only 33.7 ± 3.1% of NuSAP‐KO cells retained this pattern. Instead, 41.3 ± 3.2% of KO cells exhibited disorganized pericentrin structures (Figure 2A, lower left; Figure 2C, yellow bar), 16.3 ± 2.5% displayed more than two distinct pericentrin foci (Figure 2A, upper right; Figure 2C, orange bar), and 8.7 ± 1.2% showed both an increased number and marked disorganization of pericentrin foci (Figure 2A, lower right; Figure 2C, blue bar).

We further quantified the number of pericentrin foci as an indicator of PCM fragmentation. The majority of WT cells, with 97 ± 3% falling into this category, maintained a normal number of foci (≤2 per cell), whereas only 70.7 ± 2.3% of NuSAP‐KO cells retained this pattern (Figure 2D, green bars), indicating a substantial increase in PCM disorganization upon NuSAP loss. To assess whether these defects extend to core PCM components, we examined CEP192 [61], a key upstream factor in PCM assembly. Approximately 97.0 ± 3.0% of WT cells exhibited two well‐organized CEP192 foci during G2 phase, compared to only 74.7 ± 1.2% of NuSAP‐KO cells displaying this normal pattern (Figure S1H,I).

Together, these findings demonstrate that NuSAP depletion causes pronounced disruption of pericentrin distribution and broader PCM architecture during G2 phase.

### 2.3 Knockdown of NuSAP in Normal Cells Leads to Centriole Precocious Disengagement and PCM Disorganization

To ensure that the effects of NuSAP depletion are not specific to HeLa cells, which may have compromised cell cycle checkpoints due to their cancerous nature, and to distinguish centriole disengagement from mitotic multipolar spindles inherited from mother cells in our stable NuSAP‐KO HeLa cell line, we extended our investigation to the ARPE19 cell line, a spontaneously arising retinal pigment epithelial (RPE) cell line derived from normal eyes. NuSAP was knocked down in ARPE19 cells using shRNA, with knockdown efficiency confirmed by Western blot (Figure S1J). Importantly, ARPE19 shNuSAP cells exhibited similar phenotypes as those seen in NuSAP‐KO HeLa cells, reinforcing the generalizability of NuSAP depletion effects across different cell types. Specifically, PCM disorganization was observed in the G2 phase (Figure 2B, quantification in Figure 2C,D, right panels). This confirms that the role of NuSAP in maintaining centrosomal integrity is not restricted to cancer cells but extends to non‐tumorigenic cells, underscoring its broader physiological importance. While NuSAP depletion can cause spindle instability, the consistent increase in centriole disengagement and PCM disorganization across multiple cells in the G2 phase, prior to mitotic entry, suggests these defects arise independently of mitotic spindle fragmentation, supporting a direct role of NuSAP in regulating centrosomal organization.

Hence, together with the NuSAP‐KO HeLa cells data, the phenotypes observed in shNuSAP ARPE19 cells demonstrate a direct role for NuSAP in maintaining centriole engagement and PCM disorganization.

### 2.4 NuSAP Interacts With CEP57 via its MTBD

**FIGURE 3:**
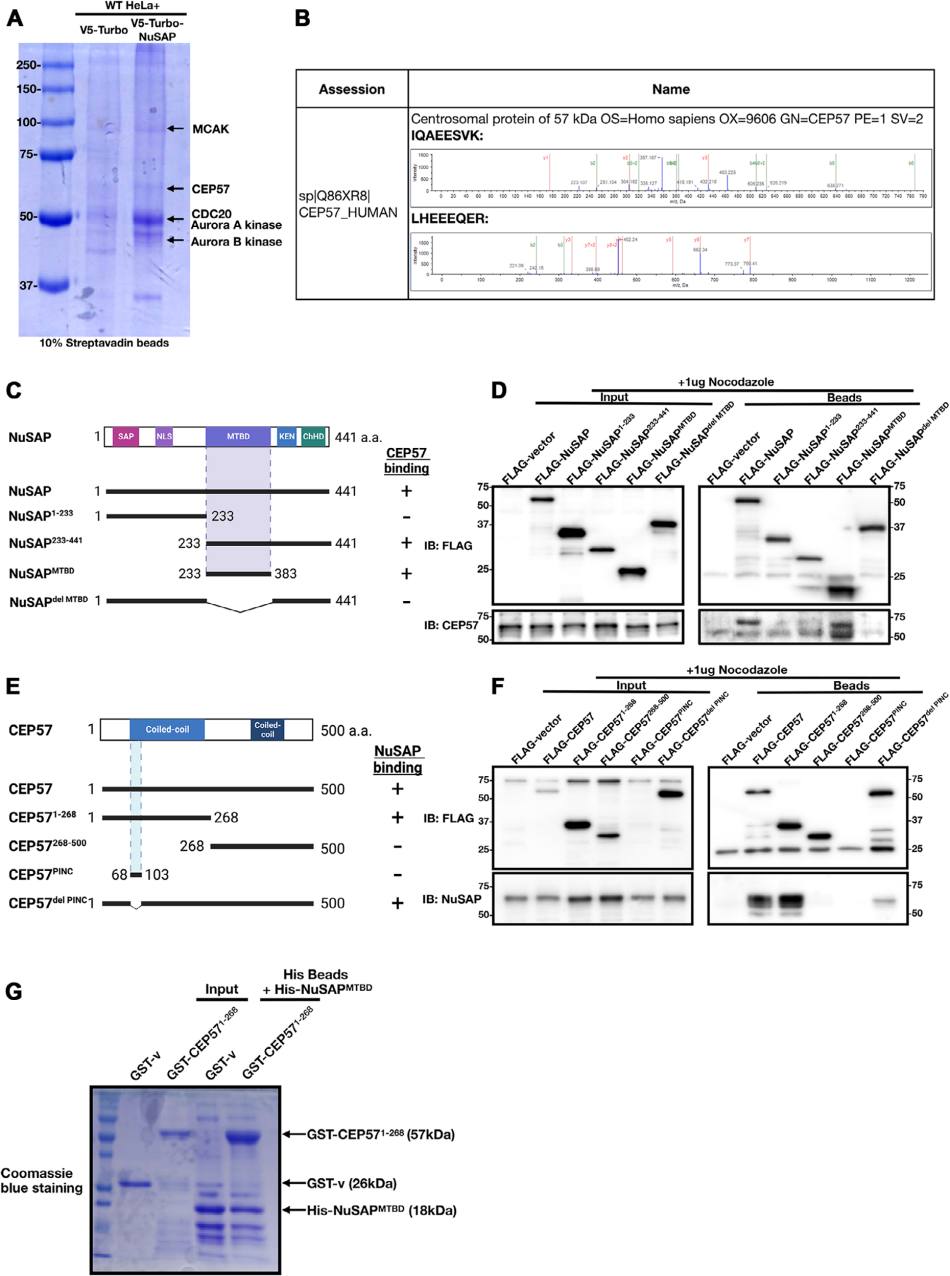
NuSAP interacts with centriole proteins, and its MTBD interacts with CEP57. (A) 10% of streptavidin beads incubated with the cell lysate cytosol of WT HeLa cells overexpressing V5‐Turbo and V5‐Turbo‐NuSAP were run in SDS‐PAGE gel and then subjected to Coomassie blue staining. Possible proteins identified are labelled. (B) Results for mass spectrometry analysis for identified protein—CEP57. (C) Schematic diagram of NuSAP and the deletion domains used for co‐IP assays. SAP, NLS, microtubule binding domain (MTBD), KEN box, and ChHD domains are shown in colored boxes. The right column shows a summary of the co‐IP results. (D) HEK293T cells overexpressing FLAG empty (control) or FLAG‐NuSAP and its domains were immunoprecipitated (IPed) with FLAG and CEP57 antibodies. 1 µg of Nocodazole was added during IP to prevent the tubulin aggregation complex, hence inaccurate IP results with the presence of microtubule binding ability. NuSAP and its MTBD interact with endogenous CEP57. (E) Schematic diagram of CEP57 and the deletion domains used for IP assays. Coiled‐coil domains were shown in colored boxes. The right column shows a summary of the IP results. (F) HEK293T cells overexpressing FLAG empty (control) or FLAG‐CEP57 and its domains were immunoprecipitated (IPed) with FLAG and NuSAP antibodies. 1 µg of Nocodazole was added. CEP57 and its N‐terminus interact with endogenous NuSAP. (G) His‐NuSAP^MTBD^, GST‐v, and GST‐ CEP57^1‐268^ were expressed and purified in BL21 competent *E. coli*, and His beads bound with His‐NuSAP^MTBD^ were used to pull down GST‐CEP57^1‐268^. Bound proteins were eluted by boiling in 2×SDS loading buffer and analyzed by SDS‐PAGE followed by Coomassie blue staining.

To investigate the mechanisms underlying NuSAP's role in maintaining centrosome integrity, we employed TurboID proximity labeling to profile proteins in close proximity to NuSAP during the S and G2 phases. As depicted in Figure 3A, our results revealed a rich interactome of NuSAP, including previously identified proteins such as Importin α and β [54, 55], CDC20 [62], KIF2C/MCAK [63], Aurora A kinase [64], and Aurora B kinase [63, 65]. Among the identified hits, the centrosomal protein CEP57 (Figure 3B) stood out due to its well‐established role in maintaining proper centriole engagement [35, 37, 42, 66].

We hypothesized that NuSAP may modulate the function of CEP57. To test this, we first sought to confirm the interaction between NuSAP and CEP57 via immunoprecipitation assays in HEK293T cells. Co‐IP assays using full‐length and various domain deletion constructs revealed that full‐length NuSAP, the C‐terminal domain (NuSAP^233‐441^), and the microtubule‐binding domain (MTBD) of NuSAP alone all interacted with CEP57 (Figure 3C,D). Since all these fragments contain the MTBD, this domain appears to be essential for NuSAP's interaction with CEP57 (Figure 3C). Consistently, depletion of the MTBD (NuSAP^del MTBD^) abolished the interaction, underscoring the essential role of the MTBD in mediating NuSAP's binding with CEP57 (Figure 3D).

To identify the specific domain of CEP57 responsible for its interaction with NuSAP, we performed reciprocal co‐IP assays using various CEP57 domain constructs (Figure 3E,F). Our results demonstrated a clear interaction between the N‐terminal region of CEP57 and NuSAP, while the C‐terminal region, which contains a centriolar localization domain, did not exhibit any interaction with NuSAP. Additionally, we explored the potential involvement of the PINC (pericentrin‐interacting) domain of CEP57 in its interaction with NuSAP. While the PINC domain alone was insufficient for the interaction, its deletion from the full‐length CEP57 significantly reduced the interaction with NuSAP, suggesting that while the PINC domain enhances the NuSAP–CEP57 interaction, it is not strictly required (Figure 3F).

To further validate that the interaction between NuSAP and CEP57 is direct, we performed in vitro pull‐down assays. His‐NuSAP^MTBD^ and GST‐CEP57^1‐268^ were expressed and purified in BL21 competent *E. coli*, and His beads bound with His‐NuSAP^MTBD^ were used to pull down GST‐CEP57^1‐268^ (Figure 3G). Our results demonstrate that His‐NuSAP^MTBD^ successfully pulled down GST‐CEP57^1‐268^, providing direct biochemical evidence of their interaction. Collectively, these results indicate the interaction of NuSAP and the centrosomal protein CEP57 and further reinforce the critical role of NuSAP in maintaining centrosome integrity.

### 2.5 NuSAP Localizes on Centrioles

**FIGURE 4:**
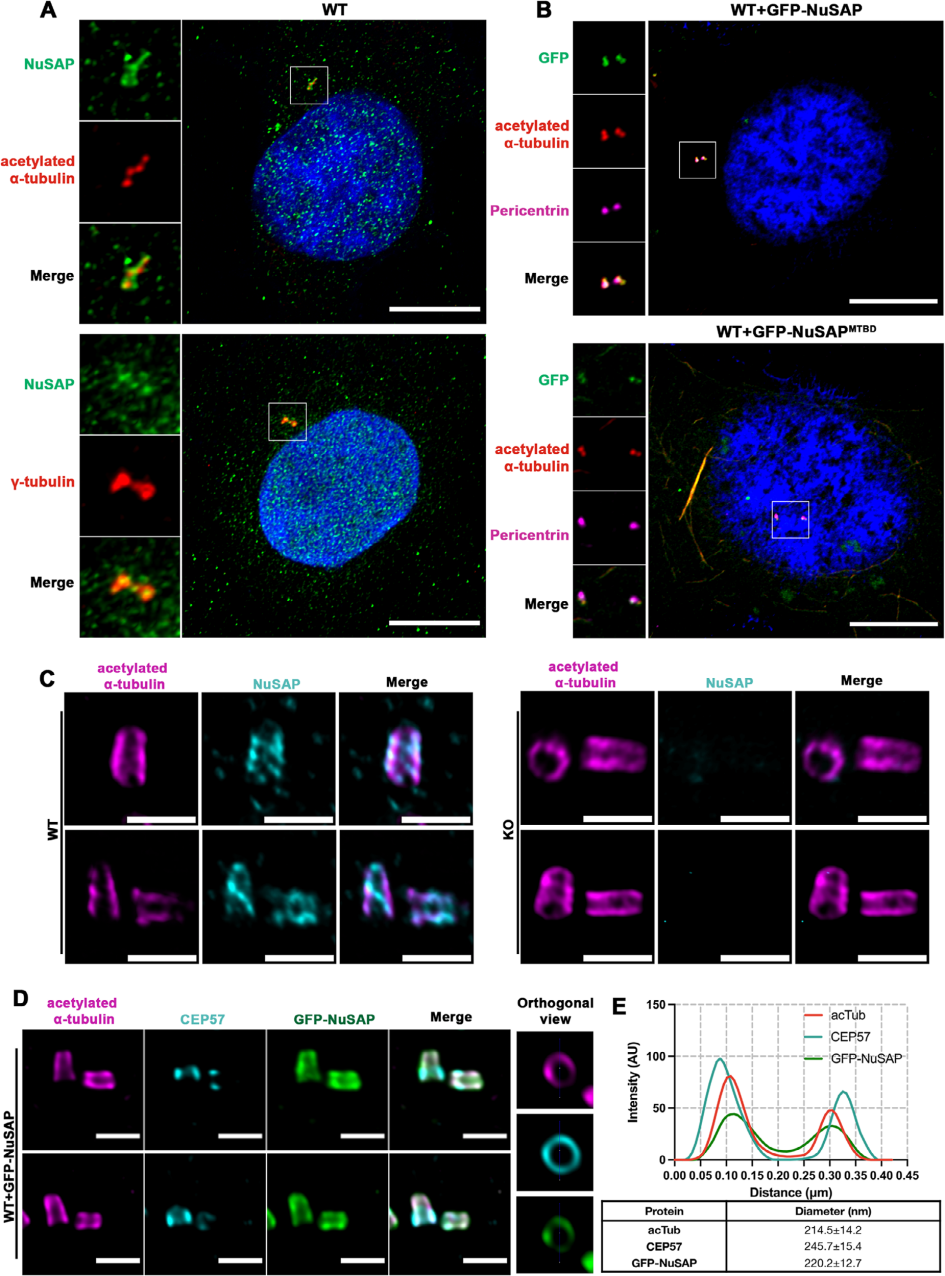
NuSAP possesses a centrosomal localization. (A) HeLa cells were synchronized in the G2 phase and stained for IF with antibodies against NuSAP (green) and (upper panel) acetylated α‐tubulin (red) with a 1‐h cold treatment before fixation to remove unstable microtubules or (lower panel) γ‐tubulin (red). Scale bar, 10 µm. (B) HeLa cells with GFP‐NuSAP and GFP‐NuSAP^MTBD^ overexpression were synchronized in the G2 phase and stained for IF with antibodies against acetylated α‐tubulin (red) and pericentrin (magenta) with a 1‐h cold treatment before fixation to remove unstable microtubules. Scale bar, 10 µm. (C) Centriolar distribution of endogenous NuSAP in the G2 phase with U‐ExM observations. HeLa cells were stained for IF with antibodies against acetylated α‐tubulin (magenta) and CEP57 (cyan), and samples were expanded according to U‐ExM preparations and observed by confocal. Scale bar, 0.5 µm. (D) Centriolar distribution of ectopic NuSAP in the G2 phase with U‐ExM observations. HeLa cells with GFP‐NuSAP overexpression were stained for IF with antibodies against acetylated α‐tubulin (magenta) and CEP57 (cyan), and samples were expanded according to U‐ExM preparations and observed by confocal. Scale bar, 0.5 µm. (E) Orthogonal views of NuSAP, CEP57, and acetylated α‐tubulin. The graph shows radial profiles from the center of NuSAP, CEP57, and acetylated α‐tubulin rings. The obtained profile was fitted with a Gaussian curve, and the distance between the center of the ring and the peak of the Gaussian curve was defined as the radius. Values are mean distance ± s.d. (n = 30).

Given that NuSAP depletion disrupts centrosome integrity and its interaction with CEP57, we hypothesized that NuSAP localizes to centrioles to exert its function. To investigate the precise localization of NuSAP, we performed conventional immunofluorescence analysis in HeLa cells using confocal microscopy (Figure 4A,B). Interestingly, endogenous NuSAP exhibited centrosomal localization regardless of cold‐treatment, as demonstrated by colocalization with acetylated α‐tubulin (cold‐treated cells, Figure 4A, upper row) and γ‐tubulin (untreated cells, Figure 4A, lower row). Nuclear NuSAP levels were greatly diminished upon cold treatment, while its localization to microtubules remains intact, reinforcing that NuSAP's association with centriole and microtubules is stable and resistant to cold treatment. The centriolar localization of NuSAP was further confirmed by overexpression of GFP‐NuSAP (Figure 4B, upper row). Moreover, overexpression of NuSAP^MTBD^ also displayed centriolar localization (Figure 4B, lower row), indicating that, besides mediating interaction with CEP57, the MTBD is responsible for NuSAP's centriolar localization.

To gain a more precise understanding of NuSAP localization on centrosomes, we employed U‐ExM. In HeLa cells, the cylindrical shape of the centriole, marked by acetylated α‐tubulin, was clearly visualized under U‐ExM (Figure 4C). We observed the procentriole growing perpendicularly from the proximal end of the mother centriole (Figure 4C). Additionally, immunostaining for NuSAP revealed a localization pattern closely aligned with acetylated α‐tubulin, with NuSAP specifically localizing along the tubulin wall of centrioles, regardless of the cell cycle phase or centriole engagement status. In the G1 phase cells, which contain one disengaged centriole pair per cell, NuSAP decorated the centriole wall (Figure 4C, upper left panel). Similarly, in the G2 phase cells, where the centrioles are in an engaged form, NuSAP remained localized with the tubulin wall of both centrioles (Figure 4C, lower left panel). No centriole localization of NuSAP was observed in NuSAP‐KO cells (Figure 4C, right panels). This consistent localization pattern across different cell cycle stages indicates a stable structural association of NuSAP with centrioles.

In addition to endogenous NuSAP, ectopically overexpressed NuSAP shows similar centriolar localization, as evidenced by the comparable signal distribution patterns observed for acetylated α‐tubulin and GFP‐NuSAP in Figure 4D. NuSAP localizes along the centriole tubulin wall and is evenly distributed throughout the centriole. Additionally, staining against CEP57 revealed a well‐assembled ring structure at the proximal end of both the mother centrioles and procentriole, consistent with previous reports [47]. Intensity line‐scan profiling of orthogonal centriole views revealed distinct ring structures for acetylated α‐tubulin, CEP57, and GFP‐NuSAP, respectively. After overlaying the intensity peaks of these three channels to investigate their relative positions and cross‐sectional diameters, the results showed that the cross‐sectional diameter of NuSAP (approximately 220.2 nm) is slightly larger than that of acetylated α‐tubulin (approximately 214.5 nm) but smaller than the CEP57 ring (approximately 245.7 nm) (Figure 4E). This spatial arrangement suggests that NuSAP is positioned neatly between the centriole tubulin wall and the CEP57 rings, supporting a model in which NuSAP directly regulates CEP57 through protein‐protein interactions, acting as a bridge between the centriole tubulin wall and the PCM, represented by CEP57.

### 2.6 NuSAP Depletion Disrupts Centriole Structure

**FIGURE 5:**
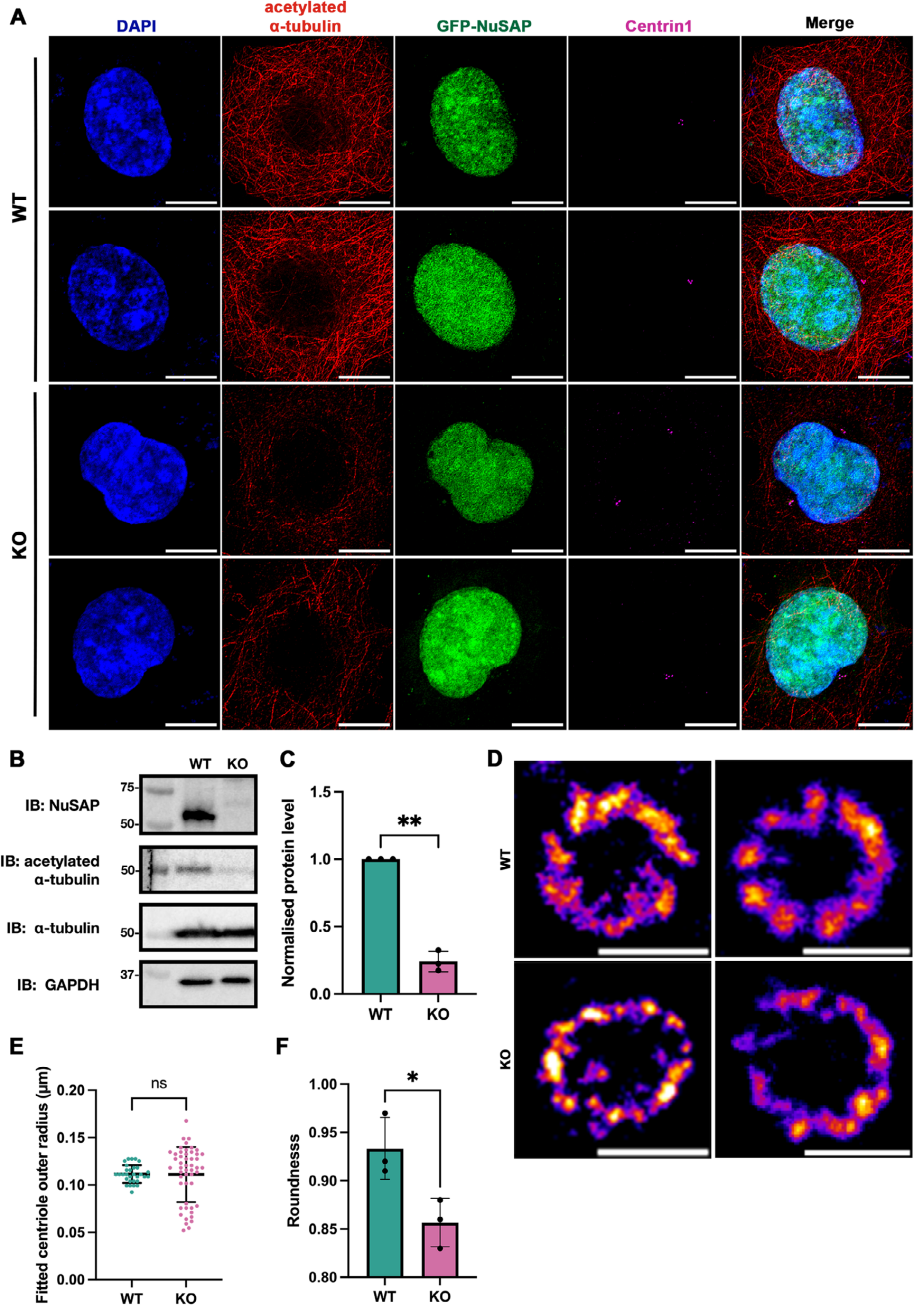
NuSAP depletion drastically reduces MT stability and induces centriole structural instability. (A) WT and NuSAP‐KO HeLa cells overexpressing GFP‐NuSAP stained for IF with antibodies against acetylated α‐tubulin (red) and Centrin1 (magenta). WT shows a higher acetylated α‐tubulin signal than KO. Scale bar, 10 µm. (B) Immunoblotting against NuSAP, acetylated α‐tubulin, α‐tubulin, and GAPDH in WT and NuSAP‐KO HeLa cells after 1‐h cold treatment. A significant decrease in acetylated α‐tubulin was observed in NuSAP‐KO cells. (C) Histograms represent the normalized acetylated α‐tubulin protein level against GAPDH in (B). Values are mean percentages ± s.d. from three independent experiments. **p < 0.01. (D) STED orthogonal views of centriole marked by acetylated α‐tubulin (fire) in WT and NuSAP‐KO cells with four times expansion. Scale bar, 0.2 µm. (E) Scattered dot plot for centriole outer radius fitted for each distinct tubulin triplet for WT and NuSAP‐KO cells (n = 27). ns = no significance. (F) Histograms represent the roundness calculated for the fitted ellipse based on each centriole orthogonal ring for WT and NuSAP‐KO cells (n = 3). *p < 0.05.

Our discovery of the strategic positioning of NuSAP between the centriole tubulin wall and the PCM motivated us to ponder its specific roles with respect to the centriole tubulin structure. Previous reports indicating the role of NuSAP in microtubule stabilization [63] spurred our interest, especially considering the substantial decrease in acetylated 𝛼‐tubulin levels observed in NuSAP‐KO cells (Figure 5A–C), signifying severe microtubule instability upon NuSAP depletion. To delve deeper, we utilized STED super‐resolution microscopy to scrutinize the structural intricacies of centriole tubulin, marked by acetylated 𝛼‐tubulin, in both WT and NuSAP‐KO cells.

In the orthogonal centriole view, WT cells displayed well‐organized tubulin structures, consisting of nine tubulin triplets (Figure 5D, first row). However, NuSAP‐KO cells exhibited disrupted centriole tubulin structures, characterized by diffused and irregular ring formations (Figure 5D, second row). Analyzing the ring structures to determine the centriole outer radius for each distinct tubulin triplet revealed no significant difference in the average fitted radius between WT and NuSAP‐KO cells. However, NuSAP‐KO cells showed significantly greater variation in the fitted centriole outer radius (0.111 ± 0.0290 µm) compared to WT cells (0.112 ± 0.0094 µm) (Figure 5E). Furthermore, quantification of the roundness of acetylated 𝛼‐tubulin rings showed a significantly lower roundness value in NuSAP‐KO centrioles (0.857 ± 0.025) compared to WT cells (0.933 ± 0.032) (Figure 5F), indicating a loss of structural regularity.

To complement these analyses, we next performed 3D reconstructions of expanded centrioles using STED super‐resolution imaging to visualize the full architecture of centriole tubulin in three dimensions. In WT cells, 3D renderings revealed a highly ordered centriole cylinder with a perfectly radial arrangement of compact tubulin triplets and a circular cross‐section (Movie S1). In contrast, NuSAP‐KO centrioles consistently displayed structural abnormalities, including discontinuities between neighboring tubulin subunits, scattered tubulin subunits, irregular or distorted ring shapes, and loss of circular symmetry, as shown in Movies S2–S4. Some NuSAP‐KO centrioles appeared oval rather than round in cross‐section (Movie S3), and multiple regions showed overt gaps within the tubulin wall. These 3D reconstructions reinforce our 2D observations, demonstrating that NuSAP is essential for maintaining the cohesive and geometrically uniform architecture of the centriole tubulin scaffold, and its depletion leads to pronounced disorganization and instability of centriole tubulin structure.

### 2.7 NuSAP is Crucial for the Precise Recruitment and Organization of CEP57 on Procentrioles

**FIGURE 6:**
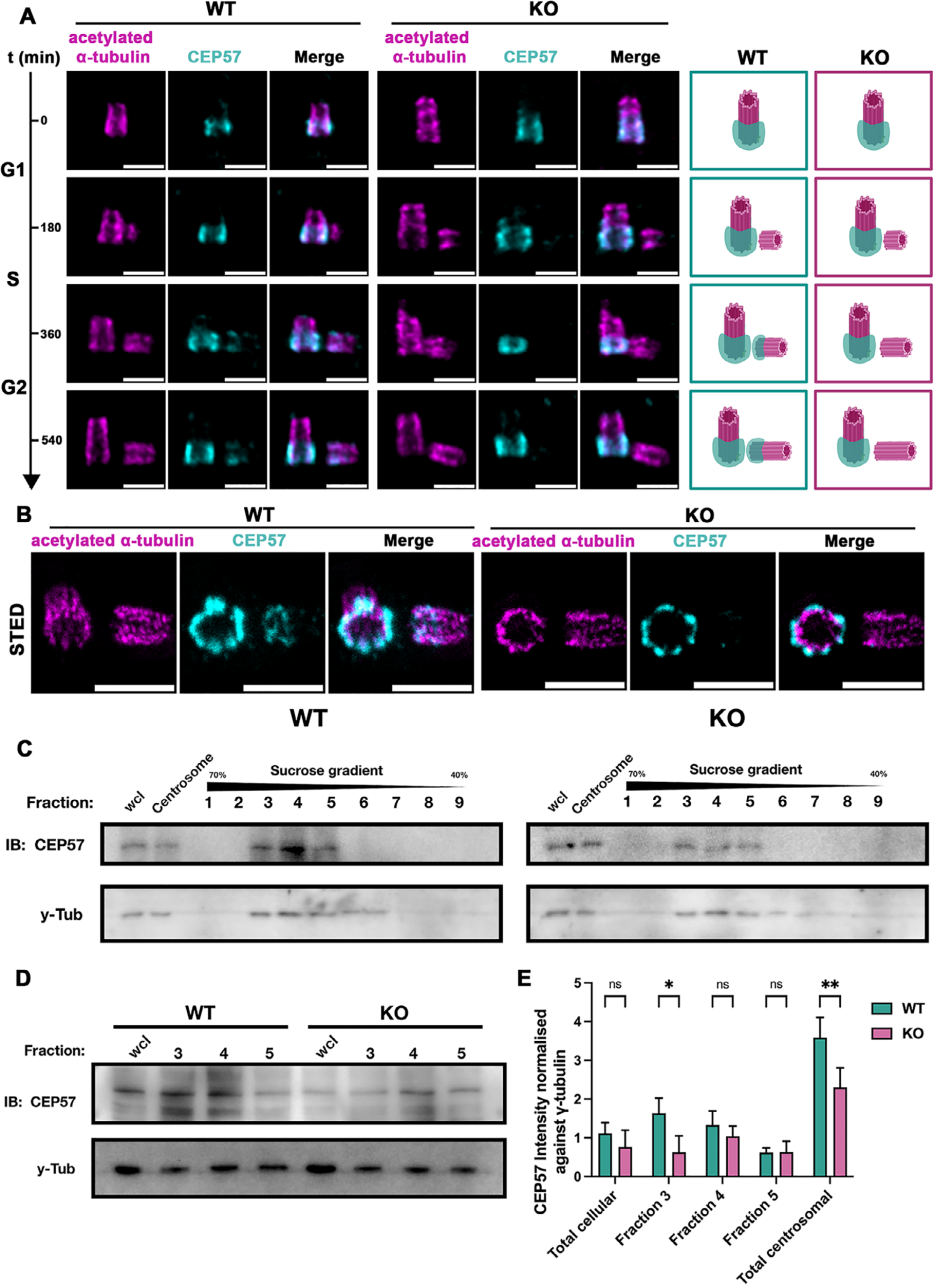
NuSAP is critical for the timely recruitment of CEP57 onto the procentriole during the S/G2 phase. (A) CEP57 becomes gradually enriched at the proximal end of procentrioles from S to G2 phase with the presence of NuSAP. The depletion of NuSAP hinders CEP57's timely recruitment onto procentrioles. WT and NuSAP‐KO HeLa cells were synchronized and released from G1/S. Cells were then fixed, expanded according to the U‐ExM protocol, and stained for IF with antibodies against CEP57 (cyan) and acetylated α‐tubulin (magenta) at every 180‐min interval until 9 h after release. Scale bar, 0.5 µm. A schematic showing CEP57 recruitment from S to G2 in both WT and NuSAP‐KO HeLa cells was attached at the right. (B) STED images acquired for centrioles marked by CEP57 (cyan) and acetylated α‐tubulin (magenta) in WT and NuSAP‐KO cells with four times expansion. Scale bar, 0.5 µm. (C) Centrosome isolation and purification in WT and NuSAP‐KO cells using 40% to 70% discontinuous sucrose gradient. Immunoblotting against CEP57 and γ‐Tubulin was carried out for whole cell lysate (wcl), centrosome fraction before being subjected to sucrose gradient separation, and 1–9 fractions collected after sucrose gradient separation for WT and NuSAP‐KO cells. (D) Immunoblotting against CEP57 and γ‐Tubulin for whole cell lysate (wcl) and 3–5 fractions collected after sucrose gradient separation for WT and NuSAP‐KO cells. (E) Histograms represent the normalized CEP57 protein level against γ‐Tubulin in (D). Values are mean percentages ± s.d. from three independent experiments. *p < 0.05. **p < 0.01. ns = no significance.

Having confirmed the interaction of NuSAP and CEP57 by the IP assay and noting that depletion of either protein leads to precocious centriole disengagement [42, 67], we hypothesized that NuSAP may regulate centriole engagement and PCM organization by modulating the functions of CEP57. To investigate this, we performed a temporal observation of CEP57 localization in WT and NuSAP‐KO cells from G1/S to G2 phases using U‐ExM. The workflow for the temporal analysis is outlined in Figure S2A. Cell‐cycle stages at each time point were validated using FACS analysis with Propidium Iodide (PI) DNA staining and immunoblotting for Cyclin B1 (Figure S2B,C). A prometaphase‐synchronized sample was included as a positive control to verify mitotic entry. Notably, Cyclin B1 levels at the final 9‐h time point remained lower than those of the prometaphase control, confirming that the cells had not yet entered mitosis. Following synchronization at the G1/S boundary with a double thymidine block (Figure 6A, top row), where two individual mother centrioles were present, CEP57 localized to the proximal end of both mother centrioles, forming a torus structure covering approximately 54% of the centriole length in both WT and NuSAP‐KO cells (Figure 6A, top row and quantification in Figure S3A,C). As cells progressed from S to G2 after 3 to 9 h release from thymidine block, procentrioles slowly grew from the proximal end of the mother centrioles (Figure 6A, second to fourth row). In WT cells, we observed a timely recruitment and enrichment of CEP57 on the proximal end of the procentriole, forming a torus that covered around 38.0% of the procentriole length in the G2 phase (Figure 6A, left panel of the last row, quantification in Figure S3D). In contrast, little or no CEP57 was detected on the procentrioles at any stage in NuSAP‐KO cells (Figure 6A, right panels, quantification in Figure S3B,D), suggesting that NuSAP is crucial for the proper recruitment and localization of CEP57 onto the procentriole.

The images acquired through STED super‐resolution microscopy (Figure 6B) aligned with our findings from conventional confocal microscopy. Collectively, these results indicate that NuSAP interacts with CEP57 and plays a critical role in the timely recruitment of CEP57 onto the procentriole during the S/G2 phase. To ensure that the regulatory role of NuSAP is not confined to HeLa cells, we examined ARPE19 shNuSAP cells and observed mis‐localization of CEP57 on the procentriole in the G2 phase (Figure S3E), reinforcing that these phenotypes represent a general cellular response to NuSAP depletion rather than being HeLa‐specific.

A comprehensive examination was conducted to assess the specific influence of NuSAP on the localization of various centriolar and PCM proteins in HeLa cells. This investigation encompassed a spectrum of proteins known for their diverse localizations and roles, including Centrin1, CEP63, CEP152, CEP192, and pericentrin (Figure S4). Remarkably, the depletion of NuSAP had no significant impact on the localization of centriolar protein Centrin1 (Figure S4A), which remained consistently localized within the upper region of the centriole lumen regardless of NuSAP presence (Figure S4A, quantification in Figure S4E).

Regarding PCM proteins, CEP192, which localizes immediately adjacent to the centriole's tubulin wall, remained consistently localized. In both WT and NuSAP‐KO cells, CEP192 consistently adhered to the centriole's tubulin wall (Figure S4B, quantification in Figure S4F). Pericentrin remained localized around the centriole pairs even after NuSAP depletion. However, despite being retained at the centrosome, the structural organization of pericentrin was significantly disrupted in NuSAP‐KO cells, as previously observed in confocal imaging (Figure 2A).

CEP57 is known to interact with CEP63 and CEP152 by forming a scaffolding complex that facilitates processes such as centriole duplication and PLK4 recruitment during S phase, and microtubule nucleation during early mitosis [35, 36, 37]. Given this, it is essential to determine whether NuSAP influences the localization of CEP63 and CEP152 in the same manner as CEP57. To address this, we conducted a localization analysis of CEP63 and CEP152. Remarkably, NuSAP depletion led to a significant reduction in the recruitment of CEP63 to the procentriole, decreasing from 88.9 ± 5.0% to 17.8 ± 5.1% (Figure S4C, quantification in Figure S4G), closely mirroring the effect observed for CEP57 (Figure 6A). Similarly, CEP152 localization was also disrupted in the same manner as CEP57 (Figure S4D, quantification in Figure S4H). These findings suggest that NuSAP plays a pivotal role in facilitating the recruitment of the CEP57‐CEP63‐CEP152 torus complex to the procentriole during the S to G2 phase, further supporting its involvement in centriole engagement and structural integrity.

Taken together, NuSAP depletion did not alter the localization of core centriolar protein Centrin1 or the immediate‐layer PCM protein CEP192. However, it significantly affected intermediate‐layer PCM proteins comprising the CEP57‐CEP63‐CEP152 torus complex. The outermost‐layer PCM protein pericentrin exhibited increased dispersal and disorganization.

### 2.8 NuSAP Depletion Reduces Centrosomal CEP57 Levels

Since NuSAP depletion abolishes CEP57 recruitment to the procentriole, we hypothesized that it could also reduce the overall centrosomal levels of CEP57. To test this, we first examined whether the total cellular protein level of CEP57 changes upon NuSAP depletion (Figure S5A). Using GAPDH as the internal control, no significant difference was observed in the total cellular protein level of CEP57 in WT and NuSAP‐KO cells. Next, to test the centrosomal CEP57 level, we isolated centrosomes from exponentially growing WT and NuSAP‐KO cells synchronized in the G2 phase. Whole‐cell lysates and centrosome‐containing supernatants were collected prior to sucrose gradient fractionation. Following ultracentrifugation, nine gradient fractions were obtained. The centrosome marker γ‐tubulin was concentrated in fractions 3 to 5, and CEP57 was also exclusively detected in these fractions in both WT and NuSAP‐KO cells (Figure 6C). When normalized to γ‐tubulin, total CEP57 levels in the centrosomal fractions were significantly reduced in NuSAP‐KO cells, with a 35.8% decrease in normalized protein level from 3.58 ± 0.52 in WT cells to 2.30 ± 0.50 in NuSAP‐KO cells (Figure 6D, quantification in Figure 6E). These findings demonstrate that NuSAP depletion specifically impairs CEP57 recruitment to centrosomes without affecting its overall expression or stability, supporting our U‐ExM observations that CEP57 recruitment to procentrioles is abolished in the absence of NuSAP.

### 2.9 NuSAP Depletion‐Induced Centriole Tubulin Structure Disruption Leads to CEP57 Mis‐Localization

As shown in Figure 5D, depletion of NuSAP resulted in significant disruption of the centriole tubulin structure. To determine whether this structural instability directly causes CEP57 mis‐localization and subsequent premature centriole disengagement, or if NuSAP acts through alternative pathways, we attempted to replicate centriole structure disruption using Vinblastine treatment. Vinblastine, previously used as a mitotic arrest agent for cell synchronization [68, 69], has been shown via electron microscopy to disrupt centriole structure and induce premature centriole disengagement [69].

**FIGURE 7:**
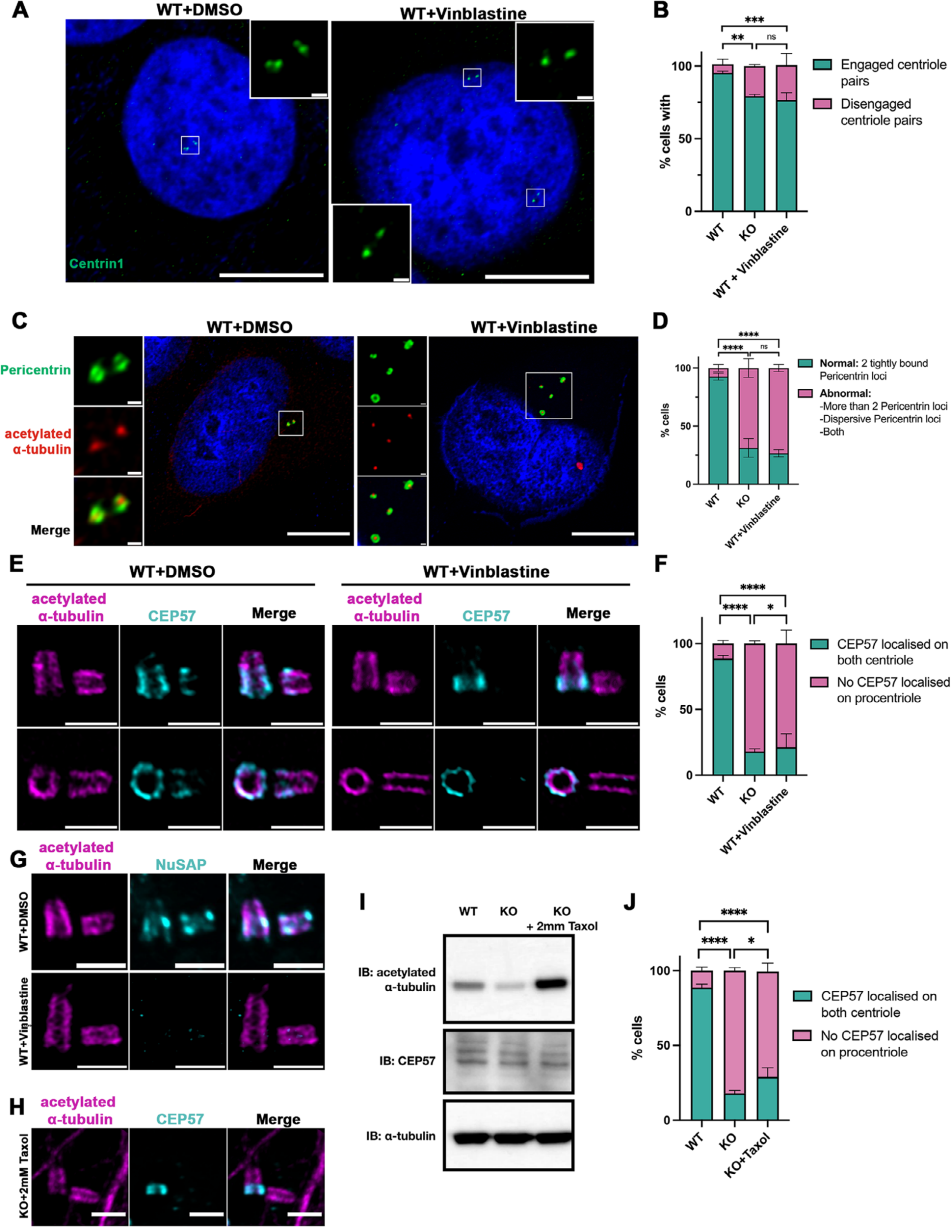
Vinblastine treatment induces precocious centriole disengagement, PCM disorganization, and CEP57 mis‐localization in WT HeLa cells. (A) WT HeLa cells were synchronized at the G1/S boundary using a double‐thymidine block (as described in Figure S2A), then released into fresh growth medium for 9 h. Cells were treated with either DMSO (control) or 10 nм Vinblastine during the final hour before collection. Cells were stained for IF with antibodies against Centrin1 (green). Scale bar, 10 µm. (B) Histograms represent the frequency of G2 cells with the dis/engaged centriole pairs in (A). Values are mean percentages ± s.d. from three independent experiments: WT n = 210, KO n = 236, WT HeLa cells treated with Vinblastine n = 224. **p < 0.01. ***p < 0.001. ns = no significance. (C) WT HeLa cells were synchronized at the G1/S boundary using a double‐thymidine block (as described in Figure S2A), then released into fresh growth medium for 9 h. Cells were treated with either DMSO (control) or 10 nм Vinblastine during the final hour. Cells were stained for IF with antibodies against acetylated α‐tubulin (red) and pericentrin (green). Scale bar, 10 µm. (D) Histograms represent the frequency of G2 cells with the indicated phenotypes observed in (C). Values are mean percentages ± s.d. from three independent experiments: WT n = 260, KO n = 291, WT HeLa cells treated with Vinblastine n = 282. *p < 0.05. ****p < 0.0001. (E) WT HeLa cells were synchronized at the G1/S boundary using a double‐thymidine block (as described in Figure S2A), then released into fresh growth medium for 9 h. Cells were treated with either DMSO (control) or 10 nм Vinblastine during the final hour. Cells were then fixed, expanded according to the U‐ExM protocol, and stained for IF with antibodies against CEP57 (cyan) and acetylated α‐tubulin (magenta). Scale bar, 0.5 µm. (F) Histograms represent the percentage of cells with or without CEP57 localization on both mother and procentrioles observed in (E). Values are mean percentages ± s.d. (three independent experiments, HeLa cells treated with DMSO n = 53, HeLa cells treated with Vinblastine n = 61). *p < 0.05. ****p < 0.0001. (G) WT HeLa cells were synchronized at the G1/S boundary using a double‐thymidine block (as described in Figure S2A), then released into fresh growth medium for 9 h. Cells were treated with either DMSO (control) or 10 nм Vinblastine during the final hour before collection. Cells were then fixed, expanded according to the U‐ExM protocol, and stained for IF with antibodies against NuSAP (cyan) and acetylated α‐tubulin (magenta). Scale bar, 0.5 µm. (H) NuSAP‐KO HeLa cells were synchronized in the G2 phase by RO3306 with an additional 2mм Taxol at the last hour. Cells were then fixed, expanded according to the U‐ExM protocol, and stained for IF with antibodies against CEP57 (cyan) and acetylated α‐tubulin (magenta). (I) Immunoblotting against acetylated α‐tubulin, α‐tubulin, and CEP57 in WT, KO, and KO treated with Taxol after a 1‐h cold treatment. (J) Histograms represent the percentage of cells with or without CEP57 localization on both mother and procentrioles observed in (H) and Figure 6A.

We treated WT HeLa cells with 10 nм Vinblastine during the final hour of the 9‐h release after double‐thymidine block to minimize Vinblastine's effects on cell cycle progression while inducing acute microtubule and centriole structure disruption [70]. The effectiveness of Vinblastine in destabilizing microtubules was confirmed by immunoblotting, as shown in Figure S5B. Immunofluorescence staining of Centrin1 revealed severe precocious centriole disengagement in the G2 phase (Figure 7A), with the percentage of cells displaying disengaged centriole pairs increasing from 6.0 ± 3.5% to 24.0 ± 8.0% (Figure 7B). Centriole disengagement has been reported to require CDK1 activity under certain conditions, particularly in the presence of DNA replication stress as report by Wilhelm et al., raising the possibility that synchronization strategies involving CDK1 inhibition may lead to an underestimation of disengagement events. Importantly, via this mechanistically distinct synchronization approach based on double thymidine block and release, which does not involve CDK1 inhibition, NuSAP depletion resulted in a comparable increase in centriole disengagement of 20.67 ± 1.16% in G2 phase cells, consistent with our observations using RO3306 (percentage of disengaged centriole pairs of 18.14 ± 4.07% in NuSAP‐KO cells) in Figure 1A,B. Together, these data suggest that although absolute disengagement levels may vary depending on synchronization strategy, the conclusion that NuSAP depletion promotes precocious centriole disengagement is robust and supported across independent synchronization.

Additionally, analysis of the PCM structure, marked by pericentrin, indicated severe PCM disorganization in the G2 phase (Figure 7C, quantification in Figure 7D). These phenotypes closely resembled those observed in NuSAP‐KO cells, suggesting that centriole tubulin disruption may be a key factor affecting CEP57 recruitment.

To test this hypothesis, we conducted a more detailed examination of CEP57 localization on the procentrioles using U‐ExM. Following Vinblastine treatment, centrioles with disrupted tubulin structures failed to recruit CEP57 onto the procentriole, resembling the phenotype seen in NuSAP‐KO cells (Figure 7E). The percentage of cells with CEP57 localized on both mother and procentrioles significantly reduced from 88.7 ± 2.3% in WT cells to 21.3 ± 10.3% following treatment (Figure 7F, first and third bars). This suggests that Vinblastine‐induced centriole structure distortion mimics the effects of NuSAP depletion on CEP57 recruitment.

Furthermore, we examined the localization of NuSAP following Vinblastine‐induced centriole microtubule destabilization. As shown in Figure 7G, NuSAP localization on both mother and procentrioles was markedly diminished or abolished upon Vinblastine treatment, indicating that centriole structural integrity is indeed critical for NuSAP recruitment.

To dissect the interdependence between NuSAP function and centriole stability, we performed a Taxol‐stabilization assay in NuSAP‐KO cells. Although Taxol treatment effectively stabilized the centriole microtubule structure, evidenced by rescued acetylated α‐tubulin protein level (Figure 7I), CEP57 localization on procentrioles remained disrupted in the absence of NuSAP. Following Taxol treatment, only 29.0 ± 6.1% of NuSAP‐KO cells showed CEP57 on both centrioles, compared to 88.7 ± 2.3% in WT cells (images in Figure 7H, quantifications in Figure 7J). These results collectively demonstrate that although centriole integrity is necessary for NuSAP localization and CEP57 recruitment and localization, NuSAP itself is a prerequisite for proper CEP57 recruitment, underscoring its pivotal role in maintaining centriole architecture and ensuring correct CEP57 positioning.

### 2.10 Rescuing Effects on PCM Disorganization by Reintroducing NuSAP and CEP57 in NuSAP‐KO Cells

To investigate whether NuSAP overexpression can mitigate the phenotypes induced by NuSAP depletion and to identify its functional domains, we reintroduced GFP‐NuSAP Full‐Length (FL), GFP‐NuSAP^MTBD^, or GFP‐NuSAP^del MTBD^ in NuSAP‐KO cells, respectively. This allows us to assess their efficacy in reversing precocious centriole disengagement and PCM disorganization phenotypes in the G2 phase.

**FIGURE 8:**
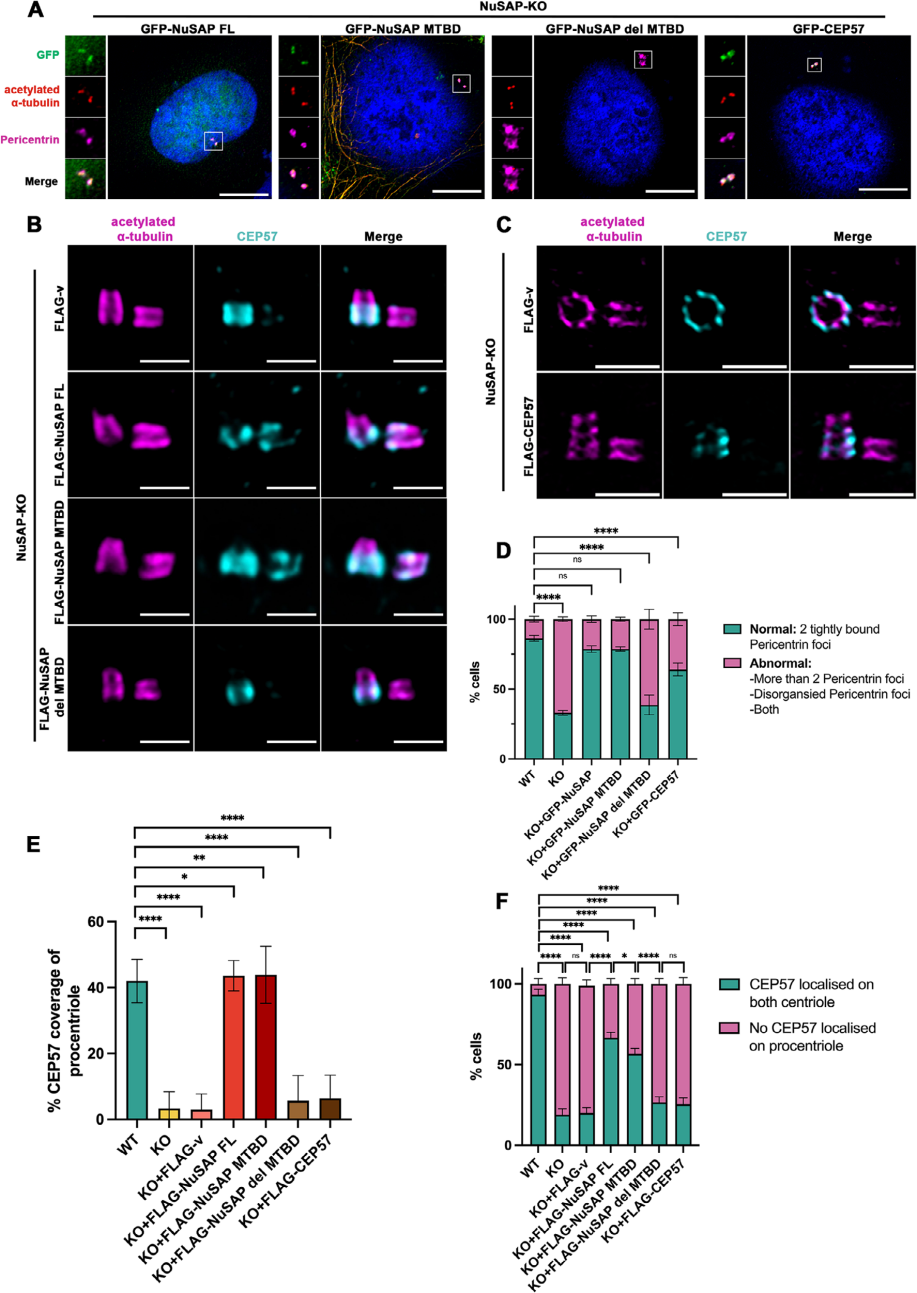
MTBD is the functional domain in NuSAP in centrosome regulation and CEP57 recruitment. (A) NuSAP‐KO HeLa cells with GFP‐NuSAP, GFP‐NuSAP^MTBD^, GFP‐NuSAP^del MTBD^, and GFP‐CEP57 overexpression were synchronized in the G2 phase and stained for IF with antibodies against acetylated α‐tubulin (red) and pericentrin (magenta). Scale bar, 10 µm. (B) NuSAP‐KO HeLa cells stably overexpressing FLAG‐v, FLAG‐NuSAP, FLAG‐NuSAP^MTBD^, and FLAG‐NuSAP^del MTBD^ overexpression were synchronized in the G2 phase and stained for IF with antibodies against acetylated α‐tubulin (magenta) and CEP57 (cyan). Samples were expanded according to U‐ExM preparations and observed by confocal. Scale bar, 0.5 µm. (C) NuSAP‐KO HeLa cells stably overexpressing FLAG‐v and FLAG‐CEP57 were synchronized in the G2 phase and stained for IF with antibodies against acetylated α‐tubulin (magenta) and CEP57 (cyan). Samples were expanded according to U‐ExM preparations and observed by confocal. Scale bar, 0.5 µm. (D) Histograms represent the frequency of G2 cells with the indicated phenotypes observed in (A). Values are mean percentages ± s.d. (three independent experiments, KO+GFP‐NuSAP n = 300, KO+GFP‐NuSAP^MTBD^ n = 300, KO+GFP‐NuSAP^del MTBD^ n = 300, and KO+GFP‐CEP57 n = 300). ns = no significance. *p < 0.05. ****p < 0.0001. (E) Histograms represent the percentage coverage of CEP57 on procentriole marked by acetylated α‐tubulin. Values are mean percentages ± s.d. (three independent experiments, KO+ FLAG‐v n = 90, KO+ FLAG‐NuSAP n = 90, KO+FLAG‐NuSAP^MTBD^ n = 90, KO+FLAG‐NuSAP^del MTBD^ n = 90, and KO+ FLAG‐CEP57 n = 90). ns = no significance. *p < 0.05. **p < 0.01. ****p < 0.0001. (F) Histograms represent the percentage of cells with or without CEP57 localization on both mother and procentrioles observed in (B) and (C). ns = no significance. *p < 0.05. ****p < 0.0001.

In the G2 phase, both GFP‐NuSAP FL (Figure 8A, the first panel) and GFP‐NuSAP^MTBD^ (Figure 8A, the second panel) effectively restored PCM organization in NuSAP‐KO cells. The percentage of cells displaying normal pericentrin structure increased to 78.7 ± 2.3% (Figure 8D, green segment in the third bar) and 78.7 ± 1.5% (Figure 8D, green segment in the fourth bar), respectively. This indicates a restoration of PCM organization compared to 86.3 ± 2.1% in WT and 33 ± 1.7% in NuSAP‐KO cells. In contrast, expression of NuSAP^del MTBD^ resulted in minimal restoration of PCM disorganization, as pericentrin still appeared disorganized and scattered (Figure 8A, the third panel), with the percentage of cells showing the normal pericentrin structure only reaching approximately 38.7 ± 7.1% (Figure 8D, green segment in the fifth bar).

Interestingly, overexpression of CEP57 only partially reversed the disorganization and the increased number of PCM foci. In G2 cells (Figure 8A, the last panel), overexpressing GFP‐CEP57 in NuSAP‐KO cells increased the percentage of cells with normal pericentrin to 64 ± 4.6% (Figure 8D, green segment in the last bar), partially restoring the PCM disorganization induced by NuSAP depletion. Thus, these results suggest that CEP57's function critically depends on the presence of NuSAP.

To determine whether NuSAP itself, and specifically its microtubule‐binding activity, is required for maintaining centriole engagement, FLAG‐NuSAP FL, NuSAP MTBD, or NuSAP del MTBD were stably expressed in NuSAP‐KO cells, and centriole engagement was analyzed by U‐ExM. Engagement status was quantified using the framework established in Figure 1C, based on the ratio (R) of the inter‐centriole distance to the mother centriole length and the relative angle between centriole pairs. Using this quantitative approach, U‐ExM imaging revealed a robust rescue of centriole engagement in NuSAP‐KO cells expressing either FLAG‐NuSAP FL or FLAG‐NuSAP^MTBD^, whereas FLAG‐v control and FLAG‐NuSAP^del MTBD^ failed to restore engagement. Specifically, the fraction of engaged centriole pairs increased to 76.30 ± 7.78% and 89.20 ± 6.23% upon expression of NuSAP FL and MTBD, respectively (Figure S5C, second and third rows; quantification in Figure S5D), comparable to WT cells (84.63 ± 4.46%). In contrast, NuSAP‐KO cells expressing FLAG‐NuSAP^del MTBD^ exhibited only 47.5 ± 2.50% engaged centriole pairs, similar to NuSAP‐KO controls (Figure S5C, last row). Together, these data demonstrate that the MTBD of NuSAP is both necessary and sufficient to restore centriole engagement and PCM disorganization in NuSAP‐KO cells.

### 2.11 The Presence of NuSAP is Essential for Anchoring CEP57 on Procentrioles

Given that NuSAP and its MTBD effectively restored centriole disengagement and PCM organization, we questioned whether they could also reinstate the recruitment of CEP57 to the proximal end of the procentriole. To evaluate their efficacy in rescuing the impaired recruitment of CEP57, FLAG‐NuSAP FL, FLAG‐NuSAP^MTBD^, and FLAG‐NuSAP^del MTBD^ were stably overexpressed in NuSAP‐KO cells.

As shown in Figure 8B, both NuSAP FL and its MTBD successfully restored the recruitment of CEP57 to procentrioles, with CEP57 coverage increasing to 41% and 44% of the procentriole length, respectively (Figure 8B, the second and third row, quantification in Figure 8E), comparable to 42% coverage observed in WT cells. Remarkably, both NuSAP FL and its MTBD also significantly restored the percentage of cells with CEP57 localized at the proximal ends of both centrioles to 66.7 ± 4.0% and 57.0 ± 3.6%, respectively (Figure 8F). In contrast, NuSAP‐KO cells stably overexpressing FLAG‐NuSAP^del MTBD^ failed to restore CEP57 recruitment (Figure 8B, last row), with only 20.7 ± 5.5% of cells exhibiting CEP57 localization on both centrioles, similar to that of NuSAP‐KO cells (Figure 8F). These findings suggest that NuSAP MTBD is an essential anchor for the CEP57 localization to the proximal end of procentrioles, and that the centriolar localization of NuSAP is crucial for governing the recruitment of CEP57. Similarly, the procentriolar localization of CEP63 and CEP152 was also restored by NuSAP FL and its MTBD (Figure S5E,F), highlighting the critical role of NuSAP and its functional centriolar domain MTBD in recruiting CEP57‐CEP63‐CEP152 torus complex.

Importantly, in the absence of NuSAP, reintroducing CEP57 alone failed to rescue its localization to the proximal end of procentrioles. Even with stable expression of FLAG‐CEP57 in NuSAP‐KO cells, CEP57 did not localize to the proximal end of procentrioles in the G2 phase (Figure 8C, the second row), with only approximately 25.5 ± 3.9% of cells exhibiting CEP57 ring localized on both centrioles, similar to NuSAP‐KO cells (Figure 8F). These findings underscore the critical role of NuSAP as an essential upstream regulator required for CEP57 recruitment and centriole torus formation.

Therefore, consolidating all the results, we can infer that the presence of functional NuSAP is critical for CEP57 recruitment at the proximal end of the procentrioles. Moreover, the NuSAP MTBD serves as the crucial functional domain responsible for the regulation of centrosome integrity by anchoring CEP57 around procentrioles, ensuring proper centriole engagement, and organizing the structure of PCM.

### 2.12 NuSAP Depletion Induces Prematuration in Precociously Disengaged Centriole and Senescence in ARPE19 Cells

NuSAP depletion in HeLa cells in the G2 phase led to precocious centriole disengagement, accompanied by premature daughter centriole maturation, as indicated by CEP164 staining (confocal images in Figure S6A and U‐ExM images in Figure S6B, quantification in Figure S6C). This premature centriole maturation may contribute to the increased cellular senescence observed in ARPE19 NuSAP‐KD cells as assessed by β‐Galactosidase activity staining (Figure S6D). The presence of premature centrioles can potentially drive centriole overduplication within a single cell cycle. In normal cells, stringent checkpoint mechanisms are activated in response to abnormalities, such as centrosome dysfunction, to prevent genomic instability. These checkpoints halt further cell division and promote entry into senescence.

As we observed mis‐localization of CEP57 on procentrioles while the centriole pairs remained engaged, we also noted the re‐localization of CEP57 onto both centrioles following disengagement. This suggests the possibility of a second recruitment mechanism for CEP57 to the procentrioles, independent of NuSAP, after centriole disengagement. To further investigate this NuSAP‐independent recruitment, both WT and NuSAP‐KO cells were synchronized in the G2 phase (Figure S6E), and only cells with four distinctly separated centrioles were examined. Consistent with previous reports by Kim et al. [24], we observed that CEP57 localization was restored on all disengaged centrioles in both WT and NuSAP‐KO cells, indicating the presence of a NuSAP‐independent mechanism regulating CEP57 recruitment to disengaged centrioles.

CEP135 has been reported as a key regulator for CEP57 torus localization following centriole disengagement [41]. To confirm this regulatory role, we knocked down CEP135 using shRNA (shCEP135) and examined CEP57 localization in disengaged centriole pairs. In line with previous findings, CEP57 localization was absent from disengaged centrioles in shCEP135 NuSAP‐KO cells (Figure S6F, lower panel, quantification in Figure S6G). In shCEP135 WT HeLa cells, we observed reduced recruitment of CEP57, further supporting the role of CEP135 in regulating CEP57 localization (Figure S6F, upper panel, quantification in Figure S6G). These findings suggest that CEP57 centriole torus recruitment is governed by distinct regulatory factors at different cell cycle stages.

## 3 Discussion

Proper centrosome assembly is crucial for mitotic spindle bipolarity and accurate chromosome segregation. In this study, we depict the critical role of NuSAP in facilitating centriole engagement and orchestrating proper organization of the PCM. Our findings indicate the indispensability of NuSAP for the timely recruitment of CEP57 to the proximal end of the procentriole during the S to G2 phase of the cell cycle, revealing a novel regulatory mechanism governing the assembly of the CEP57‐CEP63‐CEP152 torus complex. Notably, we report for the first time the centriolar localization of NuSAP, both endogenously and ectopically. This strategic positioning of NuSAP between centriole tubulin and CEP57 (Figure 4D,E) empowers NuSAP to act as a molecular bridge, translating centriole tubulin structural stability to PCM organization.

**FIGURE 9:**
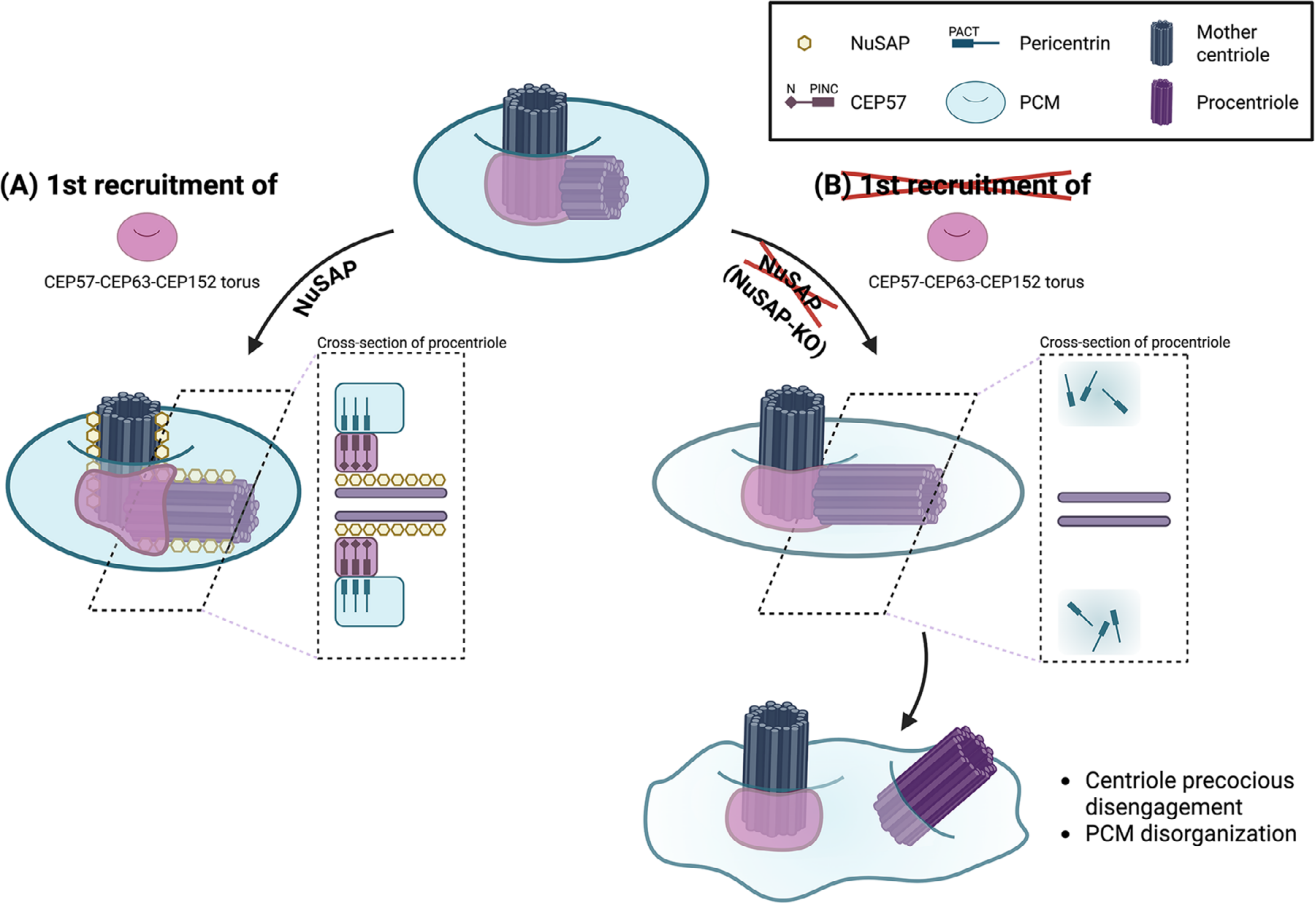
A two‐step recruitment model based on our novel findings for the CEP57‐CEP152 complex during a single cell cycle. (A) During S to G2 phase, CEP57 is gradually recruited to the procentriole, crucial for precise engagement between mother and procentrioles. This recruitment process is finely regulated by NuSAP and centriolar structural integrity. (B) In the absence of NuSAP, with centriole tubulin instability, CEP57 recruitment to procentrioles during S to G2 phase is impaired, leading to disorganized PCM structure and premature centriole disengagement. Created in BioRender. Liou, Y. (2026) https://BioRender.com/pbzrubb.

Based on our discoveries and existing studies, here we propose a novel NuSAP‐dependent recruitment model for the CEP57‐CEP63‐CEP152 torus complex. From S phase to G2, CEP57 is gradually recruited to the procentriole. This initial recruitment step is instrumental in ensuring precise engagement between the mother and procentrioles, meticulously controlled by NuSAP (Figure 9A). In the cross‐section views of procentrioles, NuSAP localizes along both mother and procentrioles via its MTBD, potentially binding to the N‐terminus of CEP57. The interaction between CEP57 and NuSAP is crucial for anchoring CEP57 to the procentriole, which in turn facilitates pericentrin localization through the binding between the PINC domain of CEP57 and the PACT domain of pericentrin [42]. This molecular interplay orchestrates the organization of a highly ordered PCM structure, ensuring the maintenance of centriole engagement during mitosis. In the absence of NuSAP, the recruitment of CEP57 to the proximal end of the procentriole is disrupted, leading to impaired organization of pericentrin and the PCM structure (Figure 9B), ultimately causing precocious centriole disengagement from G2 through mitosis.

Our biochemical mapping reveals that NuSAP binds CEP57 through its MTBD, and that CEP57 engages NuSAP primarily through its N‐terminal region with additional contribution from the PINC domain. However, the binding affinity patterns observed—specifically, the stronger interaction of isolated NuSAP MTBD compared with full‐length NuSAP (Figure 3D)—suggest additional layers of regulation beyond the presence of the binding motifs alone. One possibility is that full‐length NuSAP adopts a conformation in which the MTBD is partially masked. Intramolecular domain packing or autoinhibitory folding could limit access to the MTBD, whereas the isolated MTBD fragment remains fully exposed, thereby producing a stronger apparent interaction in pulldown assays. Similar autoinhibitory domain‐masking mechanisms have been reported for multiple microtubule‐associated and centrosomal proteins, including TPX2 [71], Kinesin‐1 [72], and Drosophila Centrosomin (Cnn) [73], where structural rearrangements or regulatory phosphorylation modulate accessibility of the functional domains. Given that NuSAP undergoes mitosis‐specific phosphorylation [74, 75], it is plausible that post‐translational modifications or microtubule binding are required to expose the MTBD in vivo.

The CEP57 side of the interaction also reveals important mechanistic nuance. Although the PINC domain is not the primary binding interface, its deletion weakens the NuSAP–CEP57 interaction. The PINC domain is known to mediate CEP57's association with pericentrin, a key component of the outer PCM scaffold [42]. This suggests that the PINC domain may help orient or stabilize CEP57 within the PCM environment, thereby indirectly promoting its interaction with NuSAP. Additionally, because CEP57 contains two coiled‐coil domains required for its liquid‐liquid phase separation (LLPS) and self‐assembly [47], removal of the PINC region—which lies within the N‐terminal coiled‐coil— could disrupt structural integrity or coiled‐coil packing, thereby altering the conformation needed for optimal NuSAP engagement. Thus, the PINC domain likely modulates the CEP57–NuSAP interface by influencing CEP57's conformational stability and/or spatial positioning within the PCM.

Collectively, these data support a model in which NuSAP binds CEP57 through an MTBD‐dependent interaction that is sensitive to protein conformation. The enhancement provided by the CEP57 PINC domain likely reflects its role in stabilizing CEP57 within the PCM or maintaining the structural configuration required for efficient NuSAP binding.

We also observed a secondary recruitment of the CEP57‐CEP63‐CEP152 torus complex to nascent procentrioles following precocious centriole disengagement in NuSAP‐KO HeLa cells (Figure S6E), demonstrating a distinct, NuSAP‐independent regulatory mechanism at different cell cycle stages. This second recruitment is orchestrated by multiple regulators, including CEP135 (confirmed in our study, Figure S6F,G), and CEP192 with CEP295 [41, 76, 77], facilitating centriole‐to‐centrosome conversion and further priming the newly formed procentrioles for subsequent centriole duplication. In the absence of NuSAP, centriole structural integrity is disrupted, retarding CEP57 recruitment to procentrioles during the S to G2 phase, leading to a disorganized PCM structure and precocious centriole disengagement. To compensate, the second recruitment of the CEP57‐CEP63‐CEP152 torus complex takes place prior to late mitosis, ensuring that disengaged procentrioles still undergo centriole‐to‐centrosome conversion, preparing them for subsequent duplication. We envisage that this meticulous interplay of the two‐step recruitment of the CEP57‐CEP63‐CEP152 complex is needed to ensure error‐free centriole duplication and maturation.

In line with our observations, recent super‐resolution studies show that the pericentriolar scaffold proteins complex CEP57, CEP63, and CEP152 form a toroidal structure at the proximal end of centrioles, with CEP63 positioned approximately 47 nm from the centriole wall, while CEP152 localizes further out at about 80 nm [39]. This structured organization supports the idea that the torus complex plays a foundational role in establishing the architecture of PCM and serves as a critical platform to recruit additional centrosomal components. Indeed, the CEP57‐CEP63‐CEP152 torus complex has long been recognized for its dual role in centriole engagement and duplication [21, 35, 36, 37, 38]. However, these processes occur at distinct cell cycle stages, raising the question of how a single complex can regulate both in a temporally coordinated manner. Centriole engagement begins in S phase and persists until mitotic exit, ensuring that procentrioles remain tethered to their mothers, which is critical for maintaining centriole integrity and preventing centriole overduplication. In contrast, centriole duplication is restricted to early S phase, requiring the precise recruitment of PLK4 to the mother centriole to initiate procentriole formation. The proposed two‐step recruitment of the torus complex provides a mechanistic framework for how it contributes to both processes. Initially, the complex localizes to the mother centriole in early S phase, where it supports centriole duplication by concentrating PLK4, ensuring efficient procentriole formation. As the cell progresses through S and G2 phases, a second wave of recruitment directs the complex to procentrioles, reinforcing centriole engagement. This stepwise assembly ensures that sufficient levels of CEP57‐CEP63‐CEP152 are available at each stage, preventing premature depletion or misallocation.

Centriole disengagement has been shown to depend in part on CDK1 activity, particularly under conditions of DNA replication stress, and synchronization strategies can influence the frequency of disengaged centrioles. In our initial analysis, cells were arrested in G2 using the CDK1 inhibitor RO3306, which allows precise staging but could potentially underestimate disengagement events. To address this, we also examined centriole engagement using a CDK1‐independent synchronization approach, in which cells were blocked at the G1/S boundary with double thymidine and released into S and G2 phases in Figure 7A,B. Importantly, NuSAP depletion produced a similar increase in centriole disengagement under this regimen, consistent with our RO3306–based observations in Figure 1A,B. Together, these results indicate that the NuSAP‐dependent disengagement phenotype is robust and not specific to a single synchronization strategy, highlighting the importance of careful cell‐cycle staging when assessing centriole architecture.

As described in Figure S6A–D, NuSAP depletion in HeLa cells in the G2 phase led to precocious centriole disengagement, accompanied by the presence of premature daughter centrioles, and contributed to the observed increase in cellular senescence in ARPE19 NuSAP‐KD cells. Indeed, disruption of core PCM components in early‐passage mouse embryonic fibroblasts (MEFs) has similarly been shown to cause centrosome fragmentation and trigger premature senescence [78]. Similarly, centrosome and microtubule integrity declines in aged human oocytes [79] and aged Drosophila cells [80], indicating that centrosome aberrations may act as cellular stressors, priming cells to exit the cell cycle permanently. These observations point to a putative link between centrosome structural abnormalities and cellular senescence. Hence, in light of our findings, further investigation on the role of NuSAP in conjunction with premature centriole maturation as a trigger of senescence could provide valuable insights into how centrosomal defects contribute to cellular aging, opening new avenues for understanding the broader implications of NuSAP function.

Moreover, our findings linking NuSAP to centrosomal functions and CEP57 scaffolding offer new insights into the potential role of *NUSAP1* in microcephaly and impaired overall development. The identification of a recurrent de novo *NUSAP1* mutation in patients with microcephaly and severe developmental delay [56], together with our functional studies demonstrating NuSAP's involvement in centrosome organization and structural integrity, underscores the clinical significance of this gene. Further mechanistic studies are crucial to determine how specific NuSAP mutations perturb centrosome dynamics and lead to neurodevelopmental disorders.

Notably, the observed expression pattern of NuSAP throughout the various cell cycle stages is reminiscent of a similar pattern observed in Drosophila syncytial blastoderm embryos. Initiation of centrosome maturation was reported to require a local pulse of Polo/PLK1 activity by centrioles before mitotic entry [81]. These findings suggestively implicate the significance of NuSAP in the process of centrosome maturation and shed light on a potential interaction between Polo and NuSAP during this process. In fact, NuSAP has been identified as an interacting partner of PLK1, through the use of PLK1's phospho‐peptide binding polo‐box domain (PBD) [82], reinforcing the hypothesis that NuSAP plays a significant role in centrosome maturation via interaction with PLK1.

Our study thus provides valuable insights into the potential functions of microtubule‐associated proteins (MAPs) in preserving the integrity of centrioles and PCM. MAPs constitute a diverse group binding to tubulin subunits, modulating microtubule stability and various cellular functions. NuSAP has been identified as a microtubule spindle stabilizer and a key regulator of chromosome oscillation [83]. Beyond its recognized functions, recent research has unveiled NuSAP's involvement in diverse cellular processes, including DNA damage repair [84], cell invasion, and metastasis [85, 86]. In this study, we further expand this understanding of NuSAP by delving into its novel regulatory roles in ensuring centrosome integrity, shedding light on the broader functions of MAPs. Our findings not only contribute to the evolving understanding of NuSAP but also inspire exploration into the multifaceted regulatory mechanisms underlying cellular processes beyond traditional boundaries.

## 4 Methods

### 4.1 Cell Culture, Cell Cycle Synchronization, and Drug Treatment

Human embryonic kidney epithelial cells (HEK 293T, RRID: CVCL_0063), human cervical carcinoma (HeLa, RRID: CVCL_0030), and spontaneously arising retinal pigment epithelial (ARPE19, RRID: CVCL_0145) were purchased from ATCC. These cells were cultured in Dulbecco's modified Eagle medium (DMEM, Sigma–Aldrich) supplemented with 10% fetal bovine serum (FBS, Gibco) at 37°C and 5% CO_2_ with 1% penicillin/streptomycin when required. The usage of all the above cell lines was approved by the NUS Institutional Review Board (NUS‐IRB‐2022‐639). HeLa and ARPE19 cell lines were authenticated by 1ST BASE ASIA on 25 March 2025. HEK293T cells were not authenticated and were used exclusively for immunoprecipitation assays to assess the interaction between NuSAP and CEP57. This interaction was further validated by an in vitro pull‐down assay performed in BL21 competent *E. coli* cells. Regular checks confirmed that HeLa, HEK293T, and ARPE19 cells were free from mycoplasma contamination, as determined by indirect testing using the DNA stain Hoechst 33342.

Cells were synchronized in the G2 phase with 9 µм RO3306 (Sigma) for 16–20 h. Subsequently, cells were washed three times with PBS before transferring to new media containing 10 µм MG132 to facilitate entry into metaphase. After 2 h, the cells were synchronized at the metaphase.

Cells were seeded to 40% confluency and synchronized at the G1/S boundary using a double‐thymidine block. Cells were treated with 2 mм thymidine for 16 h, released into fresh medium for 8 h, and subjected to a second 2mм thymidine block for 16 h. Following synchronization, cells were released into fresh growth medium and harvested every 3 h up to 9 h. For Vinblastine treatment, 10 nм Vinblastine was added during the final hour. Cells were synchronized in the G2 phase by RO3306 with an additional 2mм Taxol at the last hour.

### 4.2 Plasmids and Primers

A full‐length NuSAP was previously cloned into a pXJ40 vector with different tags, including GFP, mCherry, FLAG, or HA, respectively [63]. In addition, full‐length NuSAP and its truncation mutants, including NuSAP^MTBD^, NuSAP^1‐233^, NuSAP^233‐441^, and NuSAP^del MTBD^, were subcloned into a pXJ40 vector, each tagged with GFP, mCherry, FLAG, or HA, respectively [63]. PCRs were achieved using a Q5 High‐Fidelity DNA Polymerase. Gibson assembly was utilized for subsequent ligation.

### 4.3 Construction of NuSAP Knockout in HeLa cells

Two sgRNAs (Table 1) targeting the first exon of *NUSAP1* (Figure S1A) were created and cloned into the eSpCas9(1.1) plasmid (addgene #71814), co‐expressed with Cas9. For cell plating, a 24‐well dish with 1×10^5^ HeLa cells per well and 500 µL per well of DMEM without 10% FBS 500 was used. Using Lipofectamine 2000, 0.5 µg of the eSpCas9(1.1)‐NuSAP‐sgRNA plasmid was transfected into cells between 70% and 90% confluence. Serially diluted transfected cells (5 cells mL^−1^ medium) were plated in 96‐well plates with 100 µL of medium in each well. Only single colonies from the 96‐well plates were selected and amplified after 15 days of incubation. Both whole‐cell proteins and genomic DNAs of the selected cell clones were extracted for Western blotting and DNA sequencing, respectively, to screen for NuSAP‐knockout cells.

**TABLE 1:** Sequences of sgRNA targeting exon 1 of *NUSAP1*.

sgRNAs targeting NuSAP	Sequence (5’‐3’)
sgRNA1	Sense: GATGATCATCCCCTCTCTAG Antisense: CTAGAGAGGGGATGATCATC
sgRNA2	Antisense: CTGCAGGTCACTGTACTTGA Sense: TCAAGTACAGTGACCTGCAG

### 4.4 Transfection bPEI Transient Transfection

A 70% cell confluency was required for bPEI transient transfection. For transfection in a 10‐cm plate, 10 µg of plasmid DNA and 15 µg of bPEI (Sigma #409727) were added to 1 mL of 1× PBS solution, incubating for 20–30 min before being added to the cell culture medium.

### 4.5 Lipofectamine 2000 Transient Transfection

Lipofectamine 2000 was used at a ratio of 1.5–2 µg for every 1 µg of plasmid DNA. Before being added to the cell culture medium, Lipofectamine 2000 and plasmid were first mixed in OPTI‐MEM medium (Gibco) for 5–10 min, respectively, and then incubated for 20–30 min at room temperature. The medium was changed 8 h after incubation.

### 4.6 Stable Overexpression and Knockdown Cell Line Generation

To establish stable overexpressing V5‐TurboID‐vector, V5‐TurboID‐NuSAP, FLAG‐NuSAP, FLAG‐NuSAP^MTBD^, FLAG‐NuSAP^del MTBD^, and FLAG‐CEP57 in HeLa cells or HeLa NuSAP‐KO cells, respectively, *NUSAP1* and its domains were inserted into a lentiviral vector tagged with TurboID or FLAG. Using helper plasmids (Gag‐Pol, Rev, and VSV‐G), pLV‐cDNA vector was transfected into HEK 293T cells. After 2 days of transfection, the virus medium was collected and used to infect target cells for 48 h with 8 µg mL^−1^ of polybrene (Sigma). Stable cells were generated under the selection of 500 µg mL^−1^ G418 neomycin or 2 µg mL^−1^ puromycin.

To establish NuSAP knockdown cells in HeLa cells, the following shRNA targeting sequence was incorporated into pLV‐H1‐EF1α‐puro following the protocol from the producer, Biosettia. Similar to the procedure described above, with the help of helper plasmids (Gag‐Pol, Rev, and VSV‐G), the pLV‐H1‐EF1α‐puro vector was transfected into HEK 293T cells. After 2 days of transfection, the virus medium was collected and used to infect HeLa cells for 48 h with 8 µg mL^−1^ of polybrene (Sigma). Stable cells were generated under the selection of 2 µg mL^−1^ puromycin. Since NuSAP‐KD ARPE19 cells enter senescence shortly after transduction, a fresh batch of ARPE19 cells was generated for all relevant experiments to ensure consistency and reliability of the results.

NuSAP shRNA adopted from clone ID: TRCN0000136422. Targeting sequence: 5'‐CCTCAGGTAACAGAGATTC‐3'

CEP135 shRNA sequence: 5'‐ATAACTTGTAGAGCAAGATCTTCGC‐3'

Control LacZ shRNA sequence: 5'‐GCAGTTATCTGGAAGATCAGG‐3'

### 4.7 Antibodies

Antibodies used for immunoblotting and immunofluorescence are listed in Table 2. Antibodies were used with either 500x or 1000x dilution in 1× TBST (20 mм Tris‐HCl, 150 mм NaCl, 0.1% Tween20, pH 7.4) buffer with 0.02% sodium azide (USBIOLOGICAL).

**TABLE 2:** Antibodies.

Antibody	Host	Company	Catalog number
Anti‐FLAG	Rabbit	Sigma–Aldrich	F7425
Anti‐NuSAP	Rabbit	Proteintech	12024‐1‐AP
Anti‐CEP57	Rabbit	GeneTex	GTX115931
Anti‐GFP	Rabbit	Santa Cruz	Sc‐8334
Anti‐GAPDH	Mouse	Santa Cruz	Sc32233
Anti‐α‐tubulin	Mouse	Sigma–Aldrich	T5168
Anti‐γ‐tubulin	Mouse	Sigma–Aldrich	T5192
Anti‐acetylated α‐tubulin Anti‐pericentrin Anti‐CEP152 Anti‐CEP192 Anti‐CP110 Anti‐Centrin1 Anti‐CEP164 Anti‐CEP63	Mouse Rabbit Rabbit Rabbit Rabbit Rabbit Rabbit Rabbit	Sigma–Aldrich Abcam Bethyl Laboratories Bethyl Laboratories Proteintech Proteintech Proteintech Proteintech	T7451 Ab4448 A302‐480A A302‐324A 12780‐1‐AP 12794‐1‐AP 22227‐1‐AP 16268‐1‐AP

### 4.8 Immunoprecipitation and Western blot

For immunoprecipitation (IP), cells were washed with PBS and then harvested by NP40 lysis buffer (50 mм Tris‐HCl pH 7.4, 150 mм NaCl, 10 mм sodium pyrophosphate, and 0.4% v/v NP40) or HEPES lysis buffer (50 mм HEPES, 150 mм NaCl, 1% Triton X‐100, 10% Glycerol and 1mм EDTA at pH 7.4) for 10 min on ice. A proteasome inhibitor mixture, including 10 µg mL^−1^ aprotinin, 1 µм pepstatin A, 100 µм leupeptin, 1 mм Na_3_VO_4_, and 1 mм PMSF, was freshly added to the lysis buffer right before use. The cell lysate was applied to anti‐Flag M2 affinity gel (Sigma) for 2 h at 4°C, then washed five times with lysis buffer. Final beads were resuspended in 50 µL 2× sodium dodecyl sulphate (SDS) loading buffer at 95°C for 15–20 min. Cell lysate (input) without beads was mixed in equal amounts with 2× SDS loading buffer at 95°C for 15–20 min. For the harvest of cells directly for Western blot (WB), 2× SDS loading buffer may be directly added and placed at 95°C for at least 25 min.

The samples underwent sodium dodecyl sulfate‐polyacrylamide gel electrophoresis (SDS‐PAGE) in a running solution containing 0.1% SDS, 25 mм Tris‐HCl, and 192 mм glycine and then transferred onto a PVDF membrane (Pall Corporation) in a chilled transfer buffer. The size of the target proteins affects the transfer time, and typical transfer conditions include 100 V for 1 h or 75 V for two hours. The membrane was blocked 5% (w/v) skimmed milk in 1xTBST buffer (20 mм Tris‐HCl, 150 mм NaCl, 0.1% Tween20, pH 7.4) for 1 h at room temperature. Then the membrane was washed three times in 1xTBST buffer prior to primary antibody incubation at 4°C overnight. Subsequently, the membrane was washed three times in 1xTBST buffer prior to horseradish peroxidase (HRP)‐conjugated secondary antibodies (Sigma A4416 and A0545) incubation at room temperature for an hour. The membrane was then rinsed three times in 1xTBST buffer for 30 min each time, with a 10‐min break. Then, enhanced chemiluminescence (ECL) gel extraction was applied, and the membrane was exposed in the gel‐doc Amersham Imager 600.

### 4.9 Immunofluorescence

12‐well plates with 18 mm diameter microscope cover glasses (MARIENFELD) were used to seed cells. Following that, cells underwent cell synchronization and/or plasmid transfection when necessary. To eliminate unstable tubulin before using anti‐acetylated 𝑎‐tubulin to stain the cells for centriole imaging, cells were first placed on ice for an hour. Cells were then washed with PBS before being fixed for 7 min in ice‐cold methanol or 10 min with 1% paraformaldehyde (PFA) in methanol. After that, cells were washed three times in PBS and blocked in TBST buffer (25 mм Tris‐HCl, 137 mм NaCl, 2.7 mм KCl, 0.1% Triton X‐100, pH 7.4) containing 5% bovine serum albumin (BSA) for 1 h at room temperature.

Cells were rinsed three times with TBST before applying primary antibodies, which were diluted in 5% BSA in TBST, for 2 h at room temperature or overnight at 4°C. Thermo‐Fisher Scientific's Alexa Fluor‐conjugated secondary antibodies were then applied for 1 h at room temperature after three TBST washes separated by 5 min. The antibodies were diluted in 5% BSA in TBST buffer. Finally, Hoechst 33342 (Thermo‐Fisher) was used at 1 µg mL^−1^ for 10 min at room temperature for DNA staining. Cells were then washed at least five times with 5‐min intervals in between with TBST before mounting with FluorSave (Calbiochem) to glass slides and stored at 4°C.

### 4.10 Expansion Microscopy

The expansion microscopy protocol was adapted from Gambarotto et al. [87]. All U‐ExM samples were prepared based on this protocol, except Figure 4D, where fixation‐immunostaining was carried out before gelation to preserve GFP signals. Briefly, cells were seeded in a 12‐well plate with 13 mm diameter microscope coverslips (MARIENFELD). Cells were then treated with plasmid transfection if necessary, and/or cell synchronization. Coverslips were treated with 1.4% acrylamide (AA) – 2% formaldehyde (FA) Solution in PBS for 5 h at 37 °C to prevent protein crosslinking. A monomer solution (50 µL each 13 mm coverslip, 25 µL 38% sodium acrylate (SA), 12.5 µL 40% acrylamide (AA), 2.5 µL 2% bis‐acrylamide (BIS), 5 µL 10× PBS, 2.5 µL 10% tetramethylethylenediamine (TEMED), and 2.5 µL ammonium persulfate (APS), freshly prepared and kept at −20°C. The coverslip with monomer solution was placed on ice for 5 min before transferring to 37°C in the dark for 1 h to allow gelation. The coverslip with gel was then incubated in 4 mL denaturation buffer (200 mм SDS, 200 mм NaCl, and 50 mм Tris pH 9.0 in ddH_2_O) for 15 min at room temperature with gentle agitation. The gel was then removed from the coverslip and incubated with fresh denaturation buffer at 95°C for 90 min in a 1.5 mL Eppendorf tube.

After denaturation, the gel was then placed in a beaker with ddH_2_O for the first expansion. The water was changed at least twice every 30 min for the first two times, and it was incubated overnight. The gel was then washed with PBS twice for 30 min to remove excess water before applying primary antibodies at 4°C for 1 to 2 days. After washing with PBST (0.1% Tween‐20) several times, the gel was then incubated with secondary antibodies and Hoechst at 37°C for 2–4 h with agitation. The gel was placed in a dish with ddH_2_O for final expansion overnight. The size of the gel was measured by a caliper, and the expansion factor was calculated. The gel was cut, and excess water was removed. The cut gel was placed onto a 35 mm dish pre‐coated with poly‐L‐Lysine. A drop of ddH_2_O was added on top of the cut gel to prevent dehydration, and the side with cells was faced down, touching the glass slip.

Fixed samples were viewed under a 63×oil immersion objective on Zeiss LSM900. The wavelengths of the excitation lasers used for DAPI, GFP/ Alexa Fluor 488, mCherry/ Alexa Fluor 568, and Alexa Fluor 633 are 405, 488, 561, and 640 nm, respectively. The exposure was adjusted to achieve the best signal‐to‐noise rate.

### 4.11 TurboID Sample Preparation for Mass Spectrometry

HeLa cells stably expressing V5‐TurboID‐vector and V5‐TurboID‐NuSAP were synchronized in the G2 phase were harvested, and lysed after 3 h of 500 µм biotin treatment. 100 µL pre‐equilibrated Streptavidin MagneSphere (Promega) beads were incubated with the cell lysate for 24 h at 4°C with end‐over‐end rotation. Beads were washed three times with wash buffer A (0.1% NP‐40 in PBS) and twice with PBS. Beads were then collected using a magnetic rack.

Beads were resuspended in 500 mм pH8.5 triethylammonium bicarbonate (TEAB) with 2 mм tris(2‐carboxyethyl)phosphine (TCEP), followed by heating at 65°C for 1 h. Alkylation was then conducted using 4 mм MMTS for 15 min at room temperature. After reduction and alkylation, 12.5 ng µL^−1^ trypsin was added for overnight on‐beads digestion at 37°C. Beads were separated from the digested peptides with a filter‐spin column. The digested peptide sample was then lyophilized before subjecting it to desalting using ZipTip pipette tips (Millipore, Merck). The lyophilized sample was first reconstituted in mobile buffer A (98% H_2_O, 2% acetonitrile (ACN), and 0.05% formic acid). The ZipTip pipette tips were then activated with 100% ACN and washed twice with mobile buffer A, before adding the peptide sample. The peptides were then eluted using mobile buffer B (20% H_2_O, 80% ACN, and 0.05% formic acid) before drying using a speed vacuum. The dried sample was then reconstituted in 98% H_2_O, 2% acetonitrile (ACN), and 0.1% formic acid, and analyzed using liquid chromatography‐tandem mass spectrometry (LC‐MS/MS).

The peptide separation was carried out on an Eksigent nanoLC Ultra and ChiPLC‐nanoflex (Eksigent) in trap elute configuration. Five microliters of the sample were loaded on a 200 µm × 0.5 mm trap column at a flow rate of 3 µL per minute and eluted on an analytical 75 µm × 150 mm column. Both trap and analytical columns were made of ChromXP C18‐CL, 3 µm (Eksigent). Peptides were separated by a gradient formed by 2% ACN, 0.1% FA (mobile phase A) and 98% ACN, 0.1% FA (mobile phase B): 0% to 5% of mobile phase B in 1 min, 5% to 12% of mobile phase B in 19 min, 12% to 30% of mobile phase B in 40 min, 30% to 90% of mobile phase B in 2 min, 90% to 90% of mobile phase B in 7 min, 90% to 5% of mobile phase B in 3 min and 5% to 5% of mobile phase B in 13 min, at a flow rate of 300 nL per minute.

The MS analysis was performed on a TripleTOF 5600 system (AB SCIEX) in information‐dependent mode. The MS spectra were acquired across the mass range of 400–1250 m z^−1^ in high resolution mode (>30000) using 250 ms accumulation time per spectrum. A maximum of ten precursors per cycle were chosen for fragmentation from each MS spectrum, with a 100 ms minimum accumulation time for each precursor and dynamic exclusion for 8s. Tandem mass spectra were recorded in high sensitivity mode (resolution >15000) with rolling collision energy on adjustment.

Peptide identification and quantification were performed on the ProteinPilot 5.0 software Revision 4769 (AB SCIEX) using the Paragon database search algorithm (5.0.0.4767) for peptide identification and the integrated false discovery rate (FDR) analysis function. The data were searched against a database consisting of the UniProt human database (April 2019). The search parameters are as follows: Sample Type — Identification; Cys Alkylation — Iodoacetamide; Digestion — trypsin; Special Factors — None; Species —None. The processing was specified as follows: ID Focus—Biological Modifications; Search Effort — Thorough; Detected Protein Threshold — 0.05 (10.0%). FDR analysis was performed on the dataset, and peptides identified with a confidence interval ≥95% were taken into account.

### 4.12 Image Acquisition and Imaging Analysis

All images of the same sample group were captured under consistent conditions, including identical settings for gain, offset, exposure time, and laser settings. Using Image J (Fiji) for intensity profile and diameter measurements, statistical analyses were presented as mean ± standard deviation. The method for quantifying the radius of NuSAP, CEP57, and acetylated α‐tubulin rings in both U‐ExM and STED images followed the procedure outlined by Watanabe et al. [42]. Specifically, the ImageJ plugin “Radial Profile Extended” was used to generate a radial profile, assisting in defining the center and radius for analysis.

### 4.13 Centrosome Isolation

The progressive understanding of centrosomal microtubule‐organizing activity has driven the development and refinement of centrosome purification strategies. Early breakthroughs in microtubule biology [88] and the discovery of microtubule‐ and actin‐depolymerizing drugs [89, 90] enabled the first successful isolation of centrosomes. Building on these foundations, successive methodological innovations—including improvements introduced by Bornens and colleagues [91, 92]—have yielded protocols capable of producing highly enriched and functionally competent centrosomes. Such preparations have been instrumental for probing centrosome composition, microtubule nucleation capacity in vitro and in vivo [88, 91, 93, 94], and even centrosome duplication potential in Xenopus systems [95, 96]. Advances in biochemical extraction revealed the centrosome's relative resistance to high ionic strength but its sensitivity to chaotropic agents [97], further refining our understanding of its structural stability. These methodological frameworks also established the foundation for identifying core centrosomal components through immunofluorescence and immunoblotting with the enrichment in purified fractions [98, 99, 100, 101, 102]. Subsequent proteomic and ultrastructural studies expanded the centrosomal proteome to hundreds of components [1, 103, 104, 105, 106] and revealed fine details of centriolar architecture, including the distal lumen, cartwheel, and early duplication intermediates [107, 108, 109, 110, 111].

This method is adapted from Moudjou and Bornens [92] and consists of cell lysis using hypotonic solutions, followed by separation of cellular components on sucrose gradients and scaled down based on these two protocols [112, 113]. 10^8^ exponentially growing cells were harvested from five 15 cm^2^. Cells were then treated with Cytochalasin D and Nocodazole to depolymerize actin and microtubules, respectively, to facilitate centrosome isolation. Specifically, cells were scraped and re‐plated in 40 mL of fresh medium containing 8 µL of 10 mм Cytochalasin D and 80 µL of 5 µg mL^−1^ Nocodazole, achieving a final concentration of 10 µg mL^−1^ for each drug. The cells were incubated at 37°C for 1 h to allow effective drug action.

Cells were pelleted by centrifugation at 280 × g for 8 min, gently resuspended in 25 mL of 1× PBS, and centrifuged again. To further remove excess buffer, the pellet was resuspended in 25 mL of 0.1× PBS containing 8% sucrose and subjected to another round of centrifugation at 280 × g for 8 min. The cell pellet was then carefully resuspended in 2 mL of 0.1× PBS with 8% sucrose, and 8 mL of lysis buffer (1 mм HEPES pH 7.2, 0.5% NP‐40, 0.5 mм MgCl2, 0.1% β‐mercaptoethanol, 1 mм of PMSF, and 1 mg mL^−1^ of aprotinin, leupeptin, pepstatin) was rapidly added to ensure efficient lysis. The suspension was slowly inverted ten times to mix thoroughly and incubated on ice for 5 min.

To remove chromatin aggregates, unlysed cells, and swollen nuclei, the lysate was centrifuged at 2500 × g for 10 min. The resulting supernatant was filtered through a 03–150/50 nylon mesh to eliminate debris, and 1 м HEPES solution was added to achieve a final concentration of 10 mм HEPES, ensuring centrosome stabilization. To degrade any residual DNA, DNase I was added to a final concentration of 1 mg mL^−1^ (2 U mL^−1^), and the mixture was incubated at 4°C for 30 min with gentle mixing.

For centrosome concentration, the supernatant containing centrosomes was transferred into a 14 mL centrifuge bottle and carefully underlaid with 1.25 mL of 60% sucrose solution. The sample was then ultracentrifuged at 10,400 × g for 30 min. Following centrifugation, the supernatant was carefully aspirated, leaving 2.5–3 mL of centrosome‐enriched suspension, which contained approximately 20–25% sucrose.

Final centrosome purification was performed using a discontinuous sucrose gradient. A 5 mL ultracentrifuge tube was prepared by sequentially layering 0.3 mL of 70% sucrose, 0.3 mL of 50% sucrose, and 0.3 mL of 40% sucrose. The centrosome‐containing 20–25% sucrose fraction was delicately added on top of the gradient. The sample was then centrifuged at 29,000 rpm using SW60 Ti for 1 h to ensure optimal centrosome separation.

Post‐centrifugation, fractions were collected systematically: the top 3 × 1 mL fractions of supernatant were collected first, followed by nine additional fractions of 100 µL each from the bottom. To ensure sample integrity, aliquots from each fraction were reserved for immediate analysis, while the remaining fractions were flash‐frozen in liquid nitrogen and stored at −80°C for long‐term use. Immunoblotting for the centrosomal marker γ‐tubulin was performed to assess the efficiency of the isolation procedure. γ‐Tubulin was enriched in fractions 3–5, consistent with the expected distribution of purified centrosomes with proper downscaling and confirming successful centriole isolation [112].

### 4.14 In Vitro Pull‐Down Assay

BL21 competent *E. coli* cells were transformed with plasmids encoding His‐NuSAP^MTBD^ and GST‐CEP57^1‐268^, followed by overnight growth at 37°C in LB medium containing appropriate antibiotics. The next day, a fresh culture was inoculated at a 1:100 dilution and grown at 37°C until OD600 reached 0.6–0.8, indicating mid‐log phase growth. Protein expression was induced by adding 0.5 mм IPTG, and the culture was incubated for an additional 16 h at 20°C to facilitate proper protein folding. Cells were harvested by centrifugation at 4000 × g for 10 min at 4°C and resuspended in lysis buffer (50 mм Tris‐HCl pH 8.0, 300 mм NaCl, 10 mм imidazole, 1 mм DTT, and protease inhibitors).

For His‐NuSAP^MTBD^ purification, the cell lysate was sonicated on ice with 10‐s pulses for a total of 5 min, then centrifuged at 15,000 × g for 30 min at 4°C to remove cell debris. The supernatant was incubated with Ni‐NTA agarose beads pre‐equilibrated with lysis buffer and gently rotated at 4°C for 2 h. The beads were washed three times with wash buffer (50 mм Tris‐HCl pH 8.0, 300 mм NaCl, and 20 mм imidazole) to remove non‐specifically bound proteins. His‐tagged NuSAP^MTBD^ was left bound to the beads for direct use in pull‐down assays.

For GST‐CEP57^1‐268^ purification, bacterial lysate was prepared similarly, and the cleared supernatant was incubated with GST affinity resin (Glutathione Sepharose 4B beads) pre‐equilibrated with binding buffer (50 mм Tris‐HCl pH 8.0, 150 mм NaCl, 1 mм DTT, 0.1% Triton X‐100) for 2 h at 4°C with gentle mixing. After washing three times with wash buffer (50 mм Tris‐HCl pH 8.0, 150 mм NaCl, 1 mм DTT, 0.1% Triton X‐100, and 10 mм reduced glutathione), the bound GST‐CEP57^1‐268^ protein was eluted with 10 mм glutathione in Tris‐HCl pH 8.0 and dialyzed overnight at 4°C in interacting buffer (500mм NaCl, 50mм HEPES pH7.8, 1mм DTT, 0.5mм PMSF).

His‐NuSAP^MTBD^‐bound Ni‐NTA agarose beads were incubated with purified GST‐CEP57^1‐268^ in binding buffer at 4°C for 2 h with gentle rotation. As a control, GST‐only beads were incubated with His‐NuSAP^MTBD^ under identical conditions to assess non‐specific binding.

After incubation, beads were washed five times with interaction buffer to remove unbound proteins. Bound proteins were eluted by boiling in 2×SDS loading buffer and analyzed by SDS‐PAGE followed by Coomassie blue staining.

### 4.15 Statistical Analysis

All statistical analyses and graphics were performed using GraphPad Prism 10 software (GraphPad, La Jolla, CA, USA). Student's t test or two‐way ANOVA was used to analyze statistical significance. P < 0.05 was considered statistically significant. All data are presented as the means ± SD from at least three individual experiments. For comparing unpaired samples, a two‐tailed Student's t‐test was employed to calculate P‐values, while multiple group comparisons utilized 2‐way ANOVA.

## Author Contributions

Y.‐C.L. conceived and led the study, secured funding, and contributed to investigation, methodology, project administration, resources, supervision, and writing. S.Z. led data curation, formal analysis, investigation, methodology development, project administration, validation, visualization, and writing of the original draft. Z.J. contributed to the study conceptualization and project administration. Q.Y. contributed to funding acquisition, investigation, supervision, and project administration. H.Z. and M.W. contributed to formal analysis, resources, and visualization. C.Z. contributed to the investigation and validation. L.‐W.D. and K.C.C. provided supporting contributions to conceptualization and supervision. All authors reviewed, edited, and approved the final version of the manuscript.

## Funding

Ministry of Education Singapore Tier2 Grant A‐8000985 (Y.‐C. L)

## Conflicts of Interest

The authors declare that they have no conflicts of interest.

## Supporting information




**Supporting File 1**: advs74125‐sup‐0001‐SuppMat.docx.

**Supporting File 2**: advs74125‐sup‐0002‐Original‐blots.pdf.

**Supporting File 3**: advs74125‐sup‐0003‐MovieS1.mov.

**Supporting File 4**: advs74125‐sup‐0004‐MovieS2.mov.

**Supporting File 5**: advs74125‐sup‐0005‐MovieS2.mov.

**Supporting File 6**: advs74125‐sup‐0006‐MovieS4.mov.

## Data Availability

The data that support the findings of this study are available from the corresponding author upon reasonable request.
