# Supplementary material for: NuSAP Safeguards Centriole Integrity to Mediate CEP57‐CEP152 Torus Recruitment for Proper Engagement
Source: Adv Sci (Weinh). 2026 Jan 30;13(19):e15192. doi: 10.1002/advs.202515192 (PMC13045467; doi:10.1002/advs.202515192)
Supplement: Supplementary file 1 — Supporting File 1: advs74125‐sup‐0001‐SuppMat.docx. [file ADVS-13-e15192-s002.docx]

Supporting Information

NuSAP Safeguards Centriole Integrity to Mediate CEP57-CEP152 Torus Recruitment for Proper Engagement

*Shiyu Zhang, Zemin Jiang, Qiaoyun Yang, Hong Zheng, Minghao Wang, Chennianci Zhu, Lih-Wen Deng, Karen Carmelina Crasta, Yih-Cherng Liou**

**
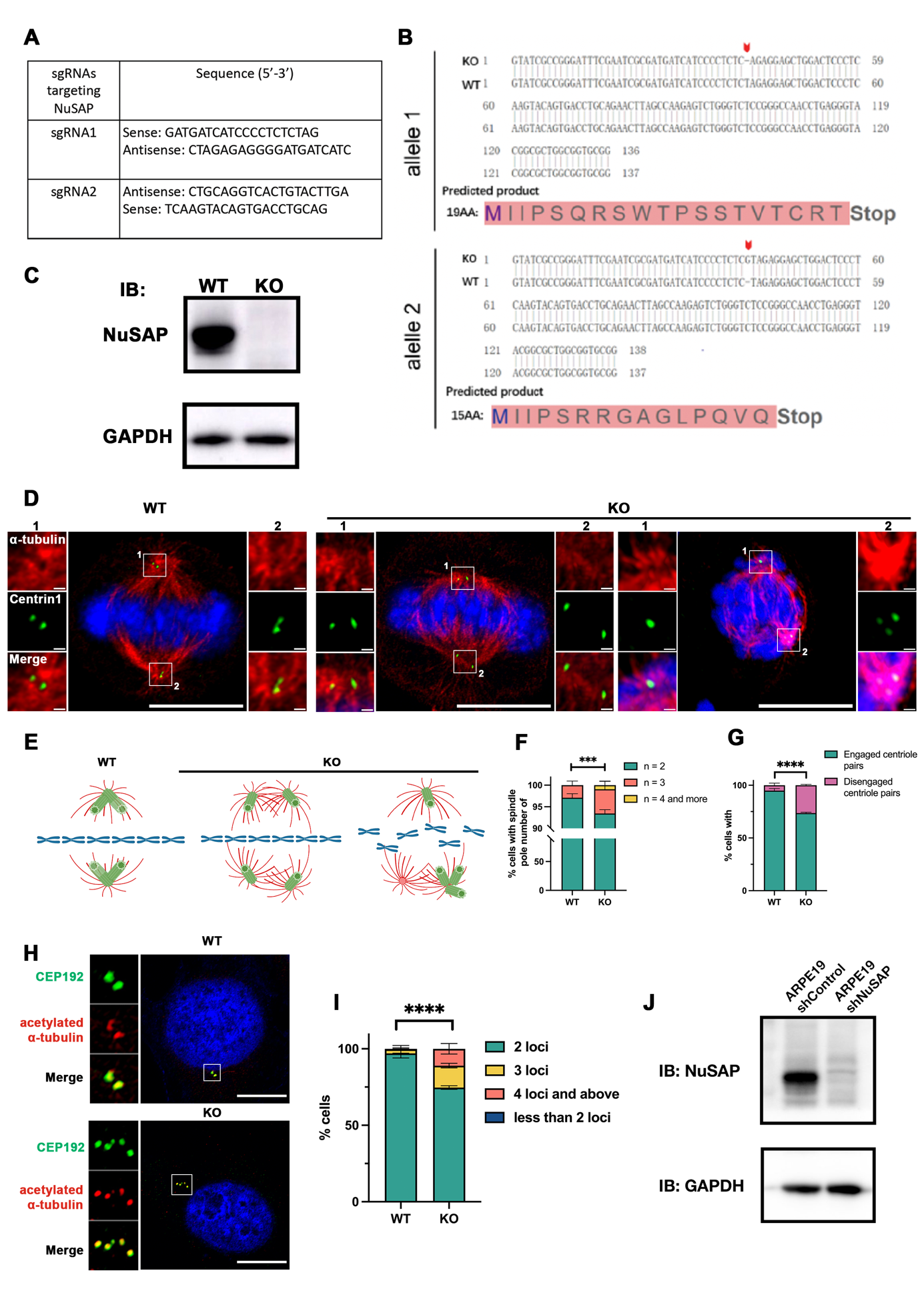
**

**Figure S1. Construction of NuSAP knockout HeLa cell lines and NuSAP knockdown ARPE19 cells**

**(A)** NuSAP-KO HeLa cell line was constructed via the CRISPR-Cas9 system. The table shows the sgRNA sequences used.

**(B)** A knockout clone with the sequence indicated, with different indel mutations (red arrows) in each allele. The first row shows a 1bp deletion in the first exon of NuSAP genomic DNA. The predicted protein product is 19 amino acids long. The second row indicates a 1bp insertion in another allele. The predicted product is 15 amino acids long.

**(C)** WT and NuSAP-KO cell lysates were collected and subjected to immunoblotting against NuSAP and GAPDH as an internal control. Complete knockout of NuSAP was observed with NuSAP-KO cells.

**(D)** WT and NuSAP-KO HeLa cells were synchronized at metaphase and stained for immunofluorescence (IF) with antibodies against α-tubulin (red) and Centrin1 (green). Scale bar, 10 μm and 0.5 μm in the inserts.

**(E)** Schematic diagram showing precociously disengaged centriole pairs.

**(F)** Histograms represent the frequency of metaphase cells with various spindle pole numbers observed in (D). Values are mean percentages ± s.d. from three independent experiments, WT n = 310, KO n = 336. ***p<0.001.

**(G)** Histograms represent the frequency of metaphase cells with dis/engaged centriole pair in **(D)**. Values are mean percentages ± s.d. from three independent experiments, WT n = 310, KO n = 336. ****p<0.0001.

**(H)** Aberrant acquisition of other PCM components was observed in NuSAP-depleted cells. WT and NuSAP-KO HeLa cells were synchronized in the G2 phase and stained for IF with antibodies against acetylated α-tubulin (red) and CEP192 (green). Scale bar, 10 μm.

**(I)** Histograms represent the frequency of G2 cells with different numbers of CEP192 foci observed in (G). Values are mean percentages ± s.d. from three independent experiments, WT n = 300, KO n = 300. ****p<0.0001.

**(J)** Immunoblotting against NuSAP and GAPDH in shControl and shNuSAP in APRE19 cells.

**
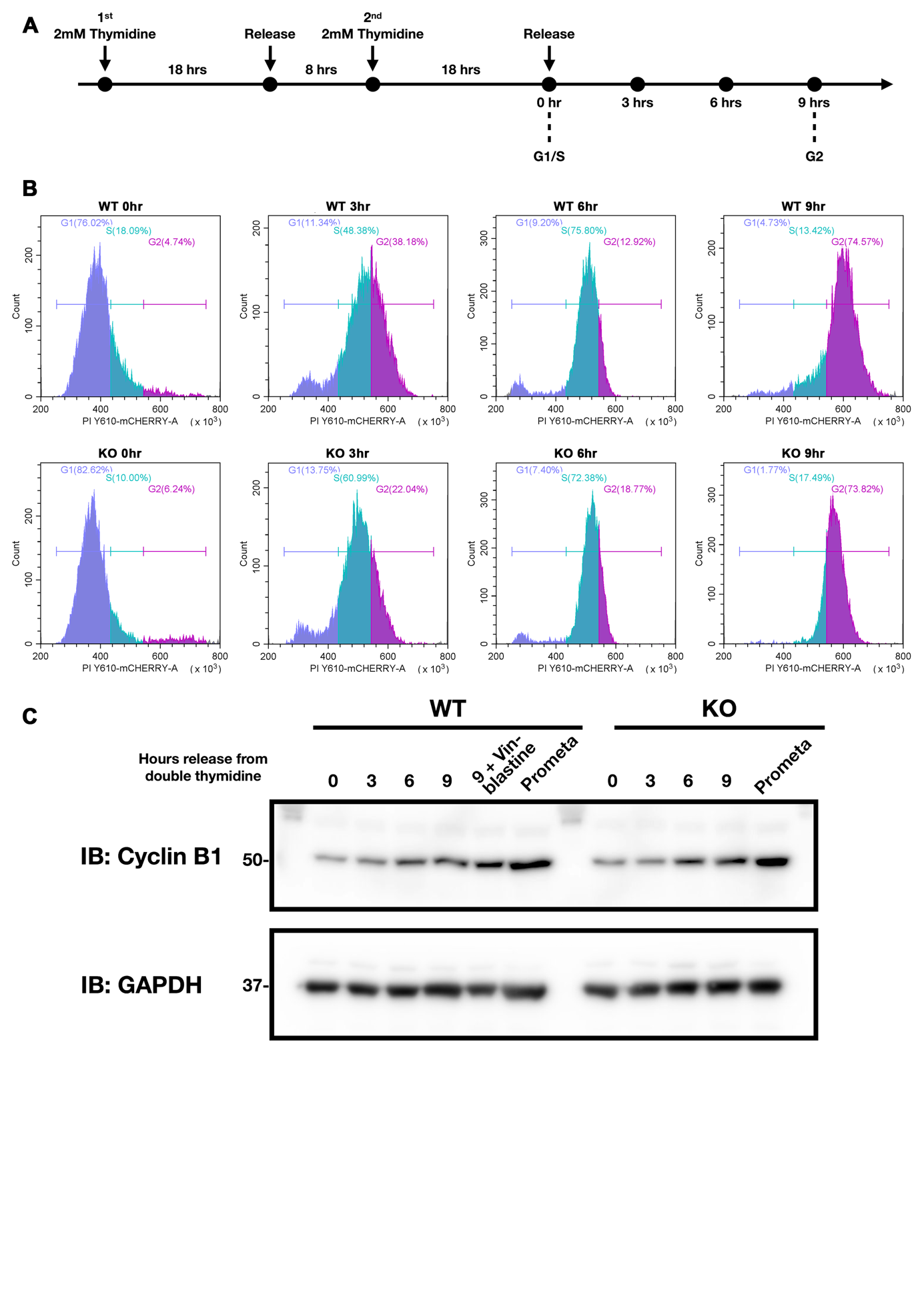
**

**Figure S2. HeLa cell cycle progression upon NuSAP depletion and temporal localization tracking of NuSAP on the centriole along cell cycle progression**

**(A)** Experimental workflow for assessing cell-cycle progression. HeLa cells were seeded to ~40% confluency and synchronized at the G1/S boundary using a double-thymidine block. Cells were treated with 2 mм thymidine for 16 hours, released into fresh medium for 8 hours, and subjected to a second 2mм thymidine block for 16 hours. Following synchronization, cells were released into fresh growth medium and harvested every 3 hours up to 9 hours for propidium iodide (PI)–based DNA content analysis to define cell-cycle stages for subsequent U-ExM assays.

**(B)** Propidium Iodide (PI) DNA-content flow cytometry profiles of WT and NuSAP-KO cells at the indicated timepoints after release from the G1/S block. Both cells progressed to G2 by 9 h after release.

**(C)** Immunoblotting against cyclin B1 and GAPDH to confirm the cell cycle stages. WT HeLa and NuSAP-KO cells were synchronized at the G1/S boundary using a double-thymidine block, then released into fresh growth medium and collected every 3 hours for 9 hours. WT cells were treated with either DMSO (control) or 10 nм Vinblastine during the final hour before collection. WT and NuSAP-KO cells were synchronized at prometaphase with 100ng/mL Nocodazole for 16 hours.

**
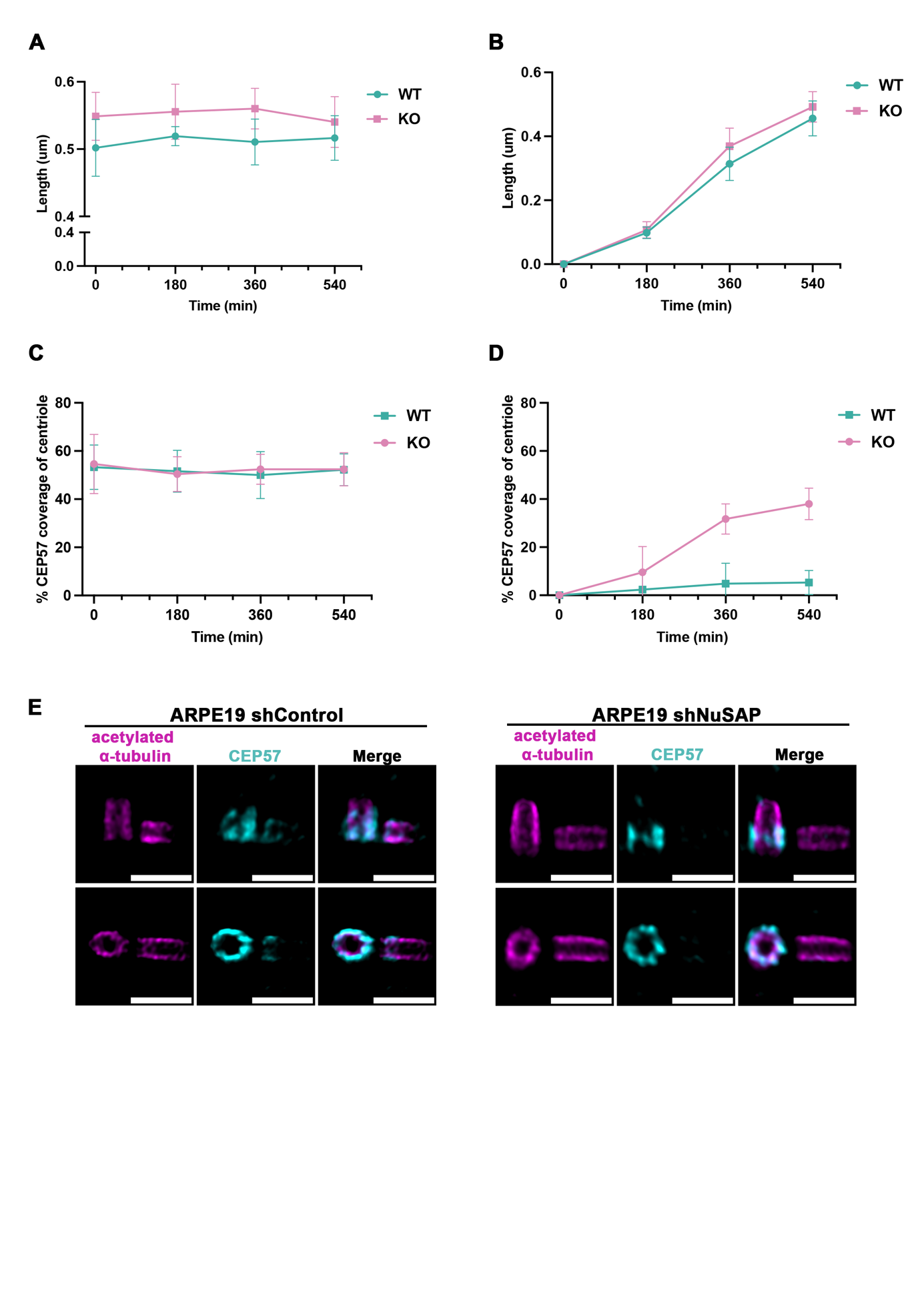
**

**Figure S3. Depletion of NuSAP abolishes the recruitment of CEP57 to the procentriole.**

**(A)** XY plots represent quantifications of mother centriole length from G1/S to G2 in WT and NuSAP-KO cells (WT n = 102, KO n=93).

**(B)** XY plots represent quantifications of procentriole length from G1/S to G2 in WT and NuSAP-KO cells (WT n = 102, KO n=93).

**(C)** XY plots represent quantification of the percentage coverage of CEP57 on mother centriole marked by acetylated α-tubulin against the time released from G1/S (WT n = 102, KO n=93).

**(D)** XY plots represent quantification of growth of the percentage coverage of CEP57 on procentriole marked by acetylated α-tubulin against the time released from G1/S (WT n = 102, KO n=93).

**(E)** shControl and shNuSAP in ARPE19 cells were synchronized in the G2 phase. Cells were then fixed, expanded according to the U-ExM protocol, and stained for IF with antibodies against CEP57 (cyan) and acetylated α-tubulin (magenta). Scale bar, 0.5 μm.

**
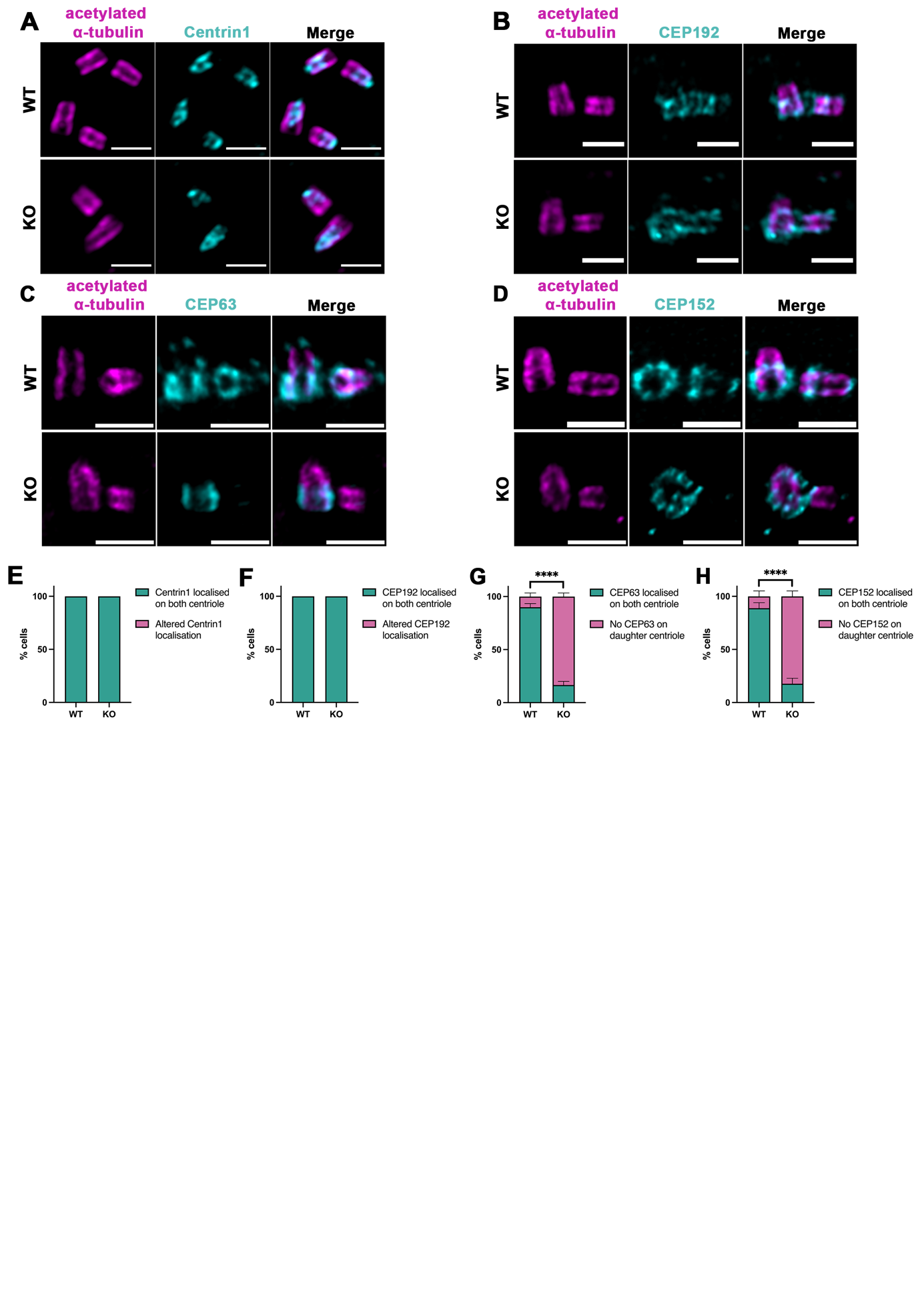
**

**Figure S4. A comprehensive assessment of the localization of various centriolar and PCM proteins upon NuSAP depletion.**

**(A-D)**WT and NuSAP-KO cells were synchronized in the G2 phase. Cells were then fixed, expanded according to the U-ExM protocol, and stained for IF with antibodies against **(A)** Centrin1 (cyan), **(B)** CEP192 (cyan), **(C)** CEP63 (cyan), **(D)** CEP152 (cyan), and acetylated α-tubulin (magenta). Scale bar, 0.5 μm.

**(E)** Histograms represent the percentage of cells with or without Centrin1 localization on both mother and procentrioles observed in (B)**.** Values are mean percentages ± s.d. (three independent experiments, WT n = 90, KO n = 90).

**(F)** Histograms represent the percentage of cells with or without CEP192 localization on both mother and procentrioles observed in (C). Values are mean percentages ± s.d. (three independent experiments, WT n = 90, KO n = 90).

**(G)** Histograms represent the percentage of cells with or without CEP63 localization on both mother and procentrioles observed in (D)**.** Values are mean percentages ± s.d. (three independent experiments, WT n = 90, KO n = 90). ****p<0.0001.

**(H)** Histograms represent the percentage of cells with or without CEP152 localization on both mother and procentrioles observed in (E). Values are mean percentages ± s.d. (three independent experiments, WT n = 90, KO n = 90). ****p<0.0001.

**
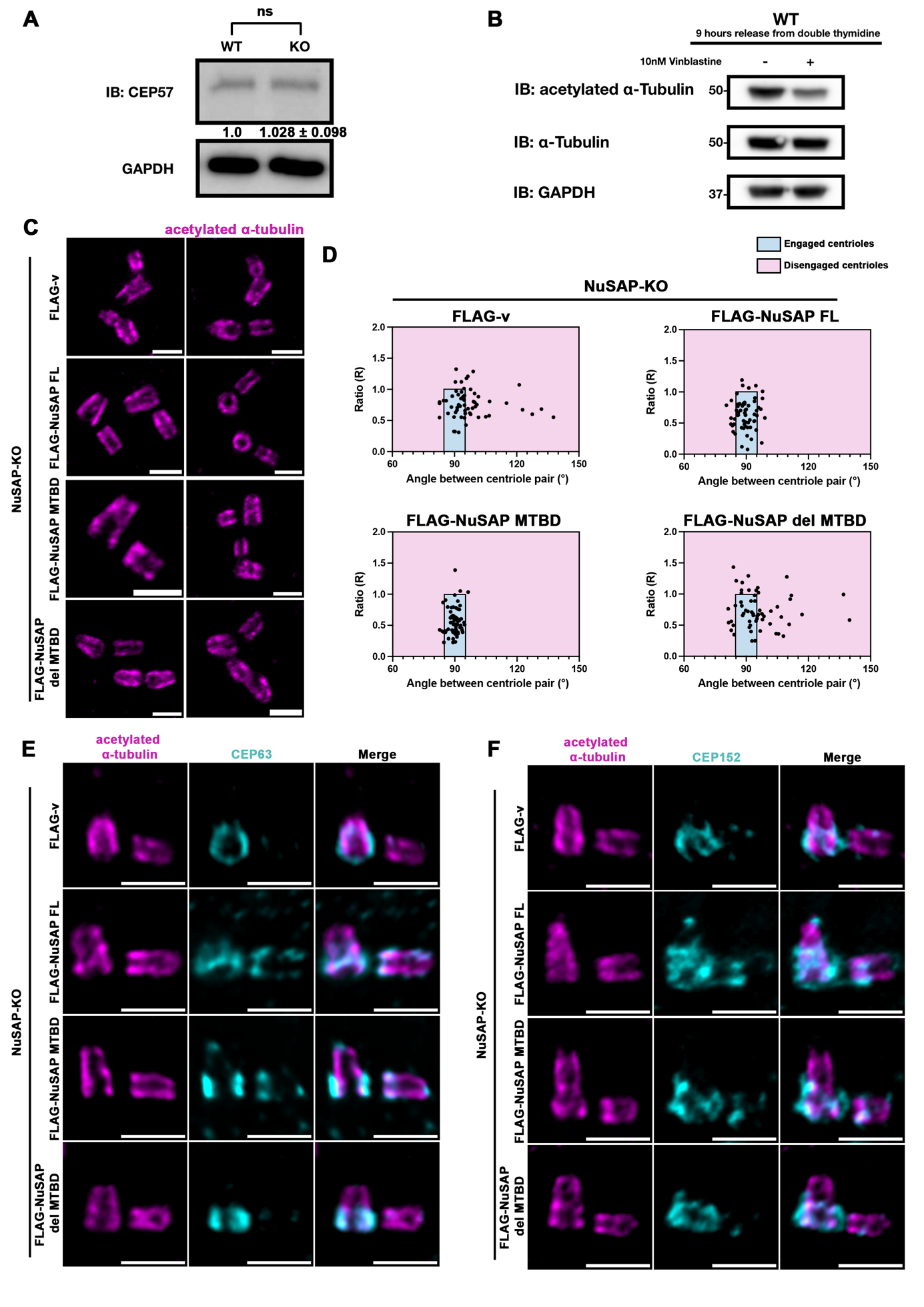
**

**Figure S5. NuSAP and its MTBD are critical for CEP63-CEP152 recruitment**

**(A)** Immunoblotting against CEP57 and GAPDH for whole cell lysate of WT and NuSAP-KO cells. Values are the mean intensity of CEP57 normalized against GAPDH ± s.d of three independent experiments. ns= no significance.

**(B)** Immunoblotting against acetylated α-tubulin, α-tubulin, and GAPDH after 1-hour cold treatment to confirm the effects of Vinblastine on microtubule stability. WT HeLa cells were synchronized at the G1/S boundary using a double-thymidine block (as described in Figure S2A), then released into fresh growth medium and collected after 9 hours. WT cells were treated with either DMSO (control) or 10 nм Vinblastine during the final hour before collection.

**(C)** U-ExM analysis of centriole configuration in NuSAP-KO HeLa cells stably overexpressing FLAG-v, FLAG-NuSAP FL, FLAG-NuSAP^MTBD^, and FLAG-NuSAP^del MTBD^ synchronized in the G2 phase. Cells were fixed, expanded according to the U-ExM protocol, and stained against acetylated α-tubulin (magenta). Scale bar, 0.5 μm.

**(D)** Dot plot quantifying centriole orientation based on the ratio of the mother–procentriole distance to mother centriole length and angle between centriole pairs. Each dot represents an individual centriole pair (FLAG-v n = 58; FLAG-NuSAP FL n = 62; FLAG-NuSAP^MTBD^ n = 63; and FLAG-NuSAP^del MTBD^ n = 60; three independent experiments).

**(E)** NuSAP-KO HeLa cells stably overexpressing FLAG-v, FLAG-NuSAP FL, FLAG-NuSAP^MTBD^, and FLAG-NuSAP^del MTBD^ were synchronized in the G2 phase and stained for IF with antibodies against acetylated α-tubulin (magenta) and CEP63 (cyan). Samples were expanded according to U-ExM preparations and observed by confocal. Scale bar, 0.5 μm.

**(F)** NuSAP-KO HeLa cells stably overexpressing FLAG-v, FLAG-NuSAP FL, FLAG-NuSAP^MTBD^, and FLAG-NuSAP^del MTBD^ were synchronized in the G2 phase and stained for IF with antibodies against acetylated α-tubulin (magenta) and CEP152 (cyan). Samples were expanded according to U-ExM preparations and observed by confocal. Scale bar, 0.5 μm.

**
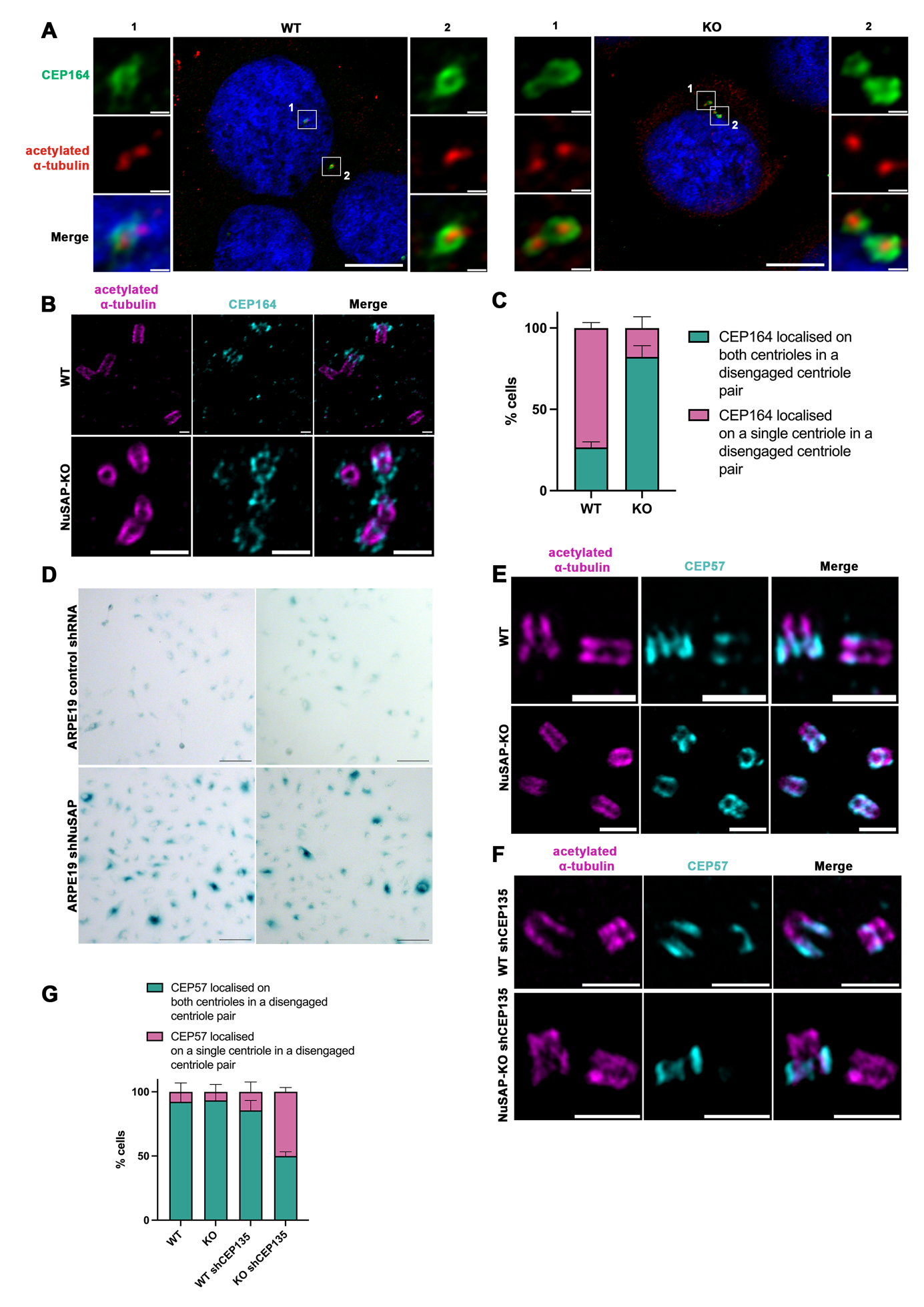
**

**Figure S6. CEP135-dependent CEP57 recruitment after centriole disengagement and pre-maturation of precocious disengaged daughter centriole induces senescence in shNuSAP in ARPE19 cells.**

(**A)** WT and NuSAP-KO HeLa cells were synchronized in the G2 phase and stained for IF with antibodies against acetylated α-tubulin (red) and CEP164 (magenta). Scale bar, 10 μm.

**(B)** WT and NuSAP-KO HeLa cells were synchronized in the G2 phase. Cells were then fixed, expanded according to the U-ExM protocol, and stained for IF with antibodies against CEP164 (cyan) and acetylated α-tubulin (magenta). Scale bar, 0.5 μm. Only cells with 4 disengaged centrioles were investigated.

**(C)** Histograms represent the percentage of cells with CEP164 localization on a single/both centriole(s) in a disengaged centriole pair observed in (B). Values are mean percentages ± s.d. (three independent experiments, WT in HeLa cells n=90, NuSAP-KO in HeLa cells n=90.)

**(D)** β-Galactosidase activity assay for shControl and shNuSAP in ARPE19 cells. Images were taken by upright fluorescence microscope.

**(E)** WT and NuSAP-KO HeLa cells were synchronized in the G2 phase. Cells were then fixed, expanded according to the U-ExM protocol, and stained for IF with antibodies against CEP57 (cyan) and acetylated α-tubulin (magenta). Scale bar, 0.5 μm. Only cells with 4 disengaged centrioles were investigated.

**(F)** shCEP135 in WT and NuSAP-KO HeLa cells were synchronized in the G2 phase. Cells were then fixed, expanded according to the U-ExM protocol, and stained for IF with antibodies against CEP57 (cyan) and acetylated α-tubulin (magenta). Scale bar, 0.5 μm. Only cells with 4 disengaged centrioles were investigated.

**(G)** Histograms represent the frequency of CEP57 localization on a single/both centriole(s) in a disengaged centriole pair observed in (E) and (F). Values are mean percentages ± s.d. (three independent experiments, WT HeLa cells n=90, NuSAP-KO HeLa cells n=90, shCEP135 in WT HeLa cells n=90, and shCEP135 in NuSAP-KO HeLa cells n=90.)

**Movie 1.** **3D reconstruction of a centriole in WT HeLa cells imaged using STEDYCON.**

Cells were fixed, expanded using the U-ExM protocol, and immunostained for acetylated α-tubulin (magenta). Scale bar corresponds to the 4× expansion. The movie shows an end-on view of the mother centriole with the procentriole positioned to its right, followed by sequential 360° rotations in the vertical and then horizontal orientations to visualize centriole architecture in three dimensions. A well-defined radial organization of centriole tubulin subunits is observed, with the procentriole extending orthogonally from the proximal end of the mother centriole.

**Movie 2. 3D reconstruction of a centriole in NuSAP-KO HeLa cells imaged using STEDYCON.**

Cells were fixed, expanded using the U-ExM protocol, and immunostained for acetylated α-tubulin (magenta). Scale bar corresponds to the 4× expansion. The movie shows an end-on view of the mother centriole with the procentriole positioned to its right, followed by sequential 360° rotations in the vertical and then horizontal orientations to visualize centriole architecture in three dimensions. The centriole shows structural defects, characterized by discontinuities between adjacent tubulin subunits and loss of the normal circular architecture.

**Movie 3. 3D reconstruction of a centriole in NuSAP-KO HeLa cells imaged using STEDYCON.**

Cells were fixed, expanded using the U-ExM protocol, and immunostained for acetylated α-tubulin (magenta). Scale bar corresponds to the 4× expansion. The movie shows an end-on view of the mother centriole with the procentriole positioned to its right, followed by sequential 360° rotations in the vertical and then horizontal orientations to visualize centriole architecture in three dimensions. Disrupted centriole tubulin organization is observed, including an oval-shaped mother centriole cross-section and breakage between neighboring tubulin subunits.

**Movie 4. 3D reconstruction of a centriole in NuSAP-KO HeLa cells imaged using STEDYCON.**

Cells were fixed, expanded using the U-ExM protocol, and immunostained for acetylated α-tubulin (magenta). Scale bar corresponds to the 4× expansion. The movie shows an end-on view of the mother centriole with the procentriole positioned to its right, followed by sequential 360° rotations in the vertical and then horizontal orientations to visualize centriole architecture in three dimensions. Clear defects in centriole tubulin organization are observed, including gaps between adjacent tubulin subunits and disruption of the normal circular architecture.
