# Supplementary material for: NuSAP Safeguards Centriole Integrity to Mediate CEP57‐CEP152 Torus Recruitment for Proper Engagement
Source: Adv Sci (Weinh). 2026 Jan 30;13(19):e15192. doi: 10.1002/advs.202515192 (PMC13045467; doi:10.1002/advs.202515192)
Supplement: Supplementary file 2 — Supporting File 2: advs74125‐sup‐0002‐Original‐blots.pdf. [file ADVS-13-e15192-s003.pdf]

Figure 3D

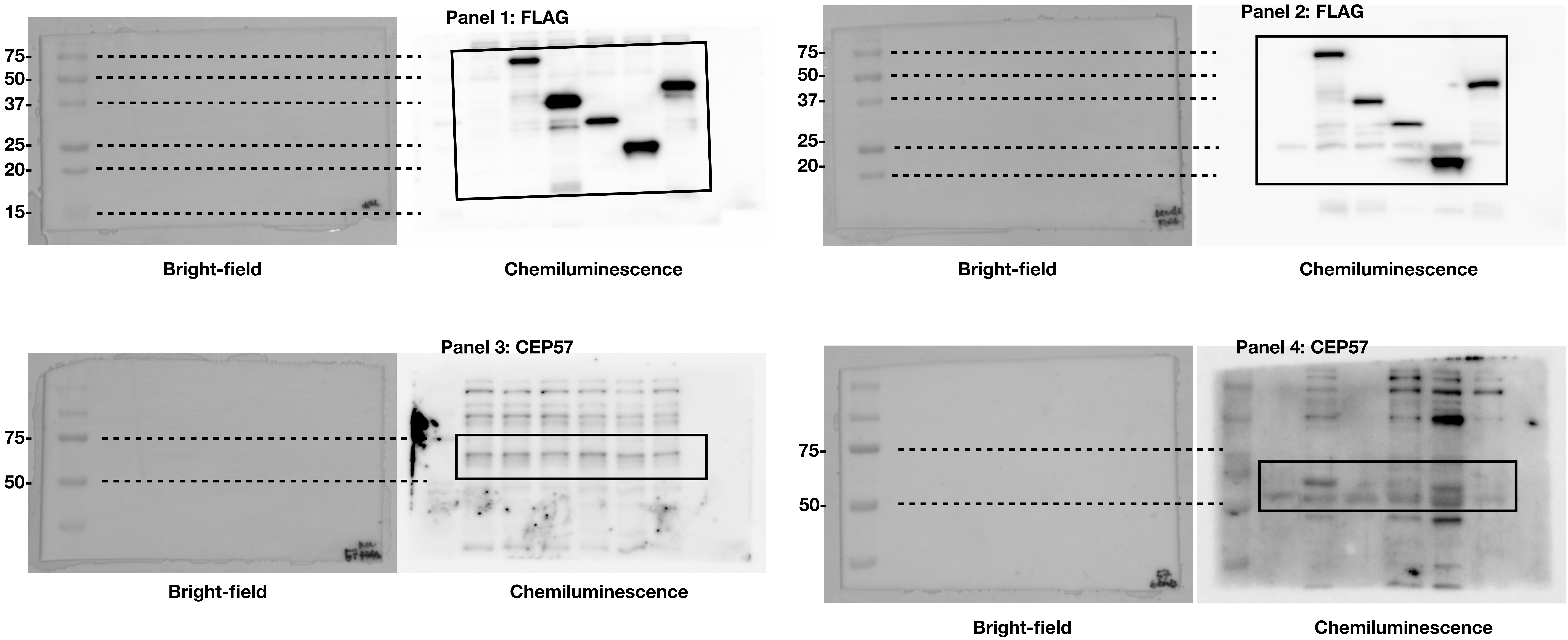

All blots were imaged using an Amersham Imager 600. Incremental exposure mode was used to capture chemiluminescence signals for the four blots. Bright-field images of the molecular weight ladder were acquired using manual mode with a 0.1 s exposure time.

Figure 3F

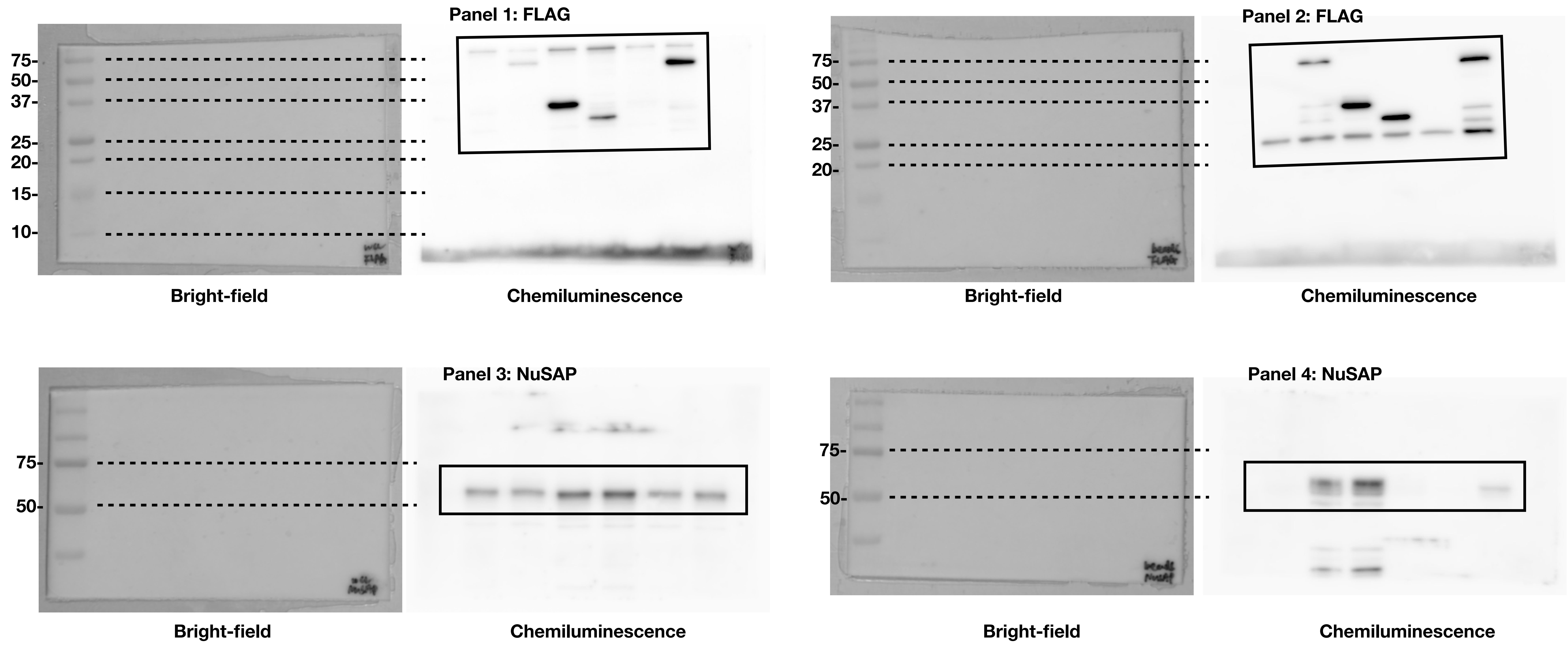

All blots were imaged using an Amersham Imager 600. Incremental exposure mode was used to capture chemiluminescence signals for the four blots. Bright-field images of the molecular weight ladder were acquired using manual mode with a 0.1 s exposure time.

Figure 5B

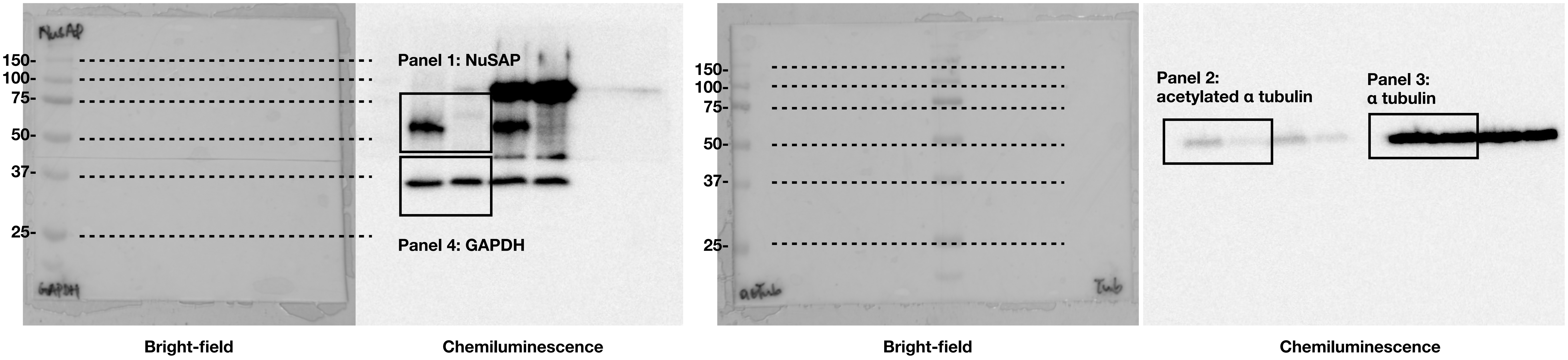

All blots were imaged using an Amersham Imager 600. Incremental exposure mode was used to capture chemiluminescence signals for the two blots. Bright-field images of the molecular weight ladder were acquired using manual mode with a 0.1 s exposure time.

Figure 6C

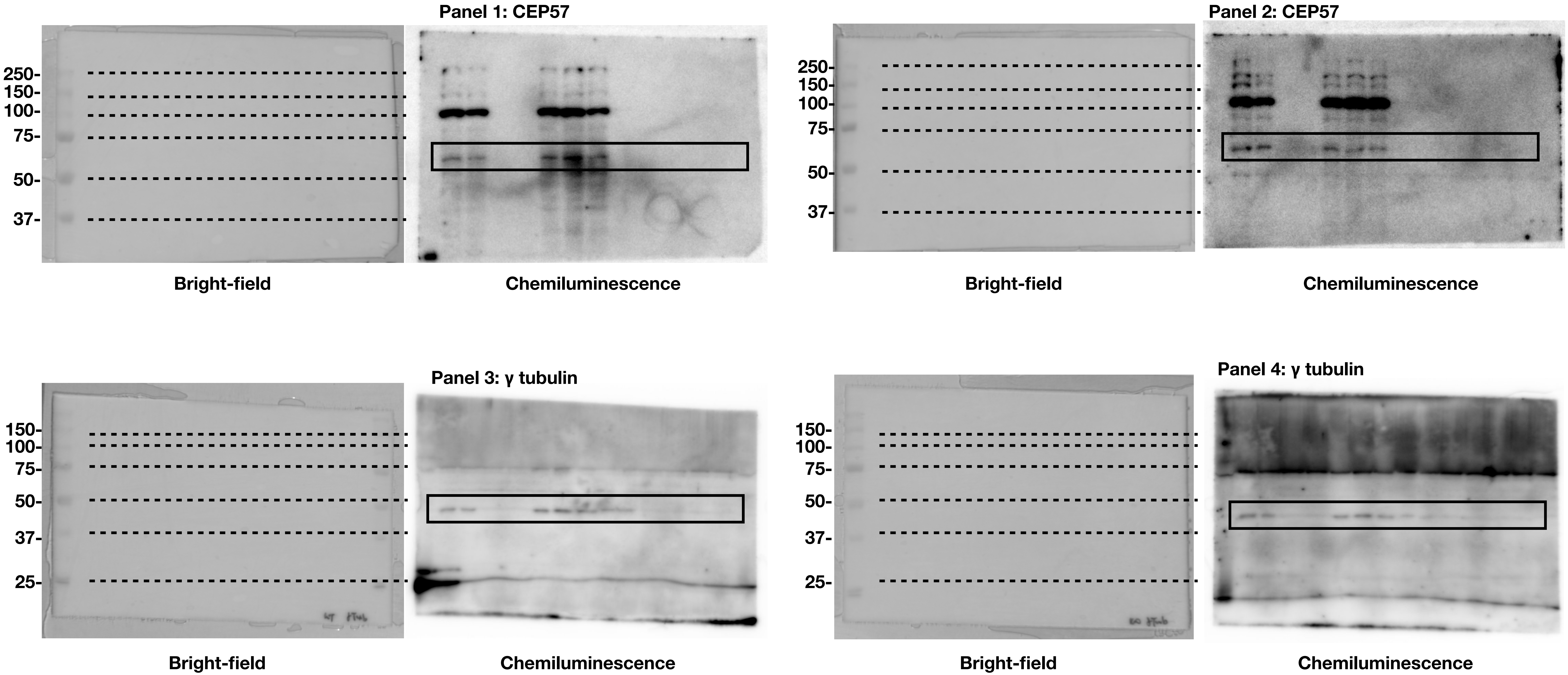

All blots were imaged using an Amersham Imager 600. Incremental exposure mode was used to capture chemiluminescence signals for the four blots. Bright-field images of the molecular weight ladder were acquired using manual mode with a 0.1 s exposure time.

Figure 6D

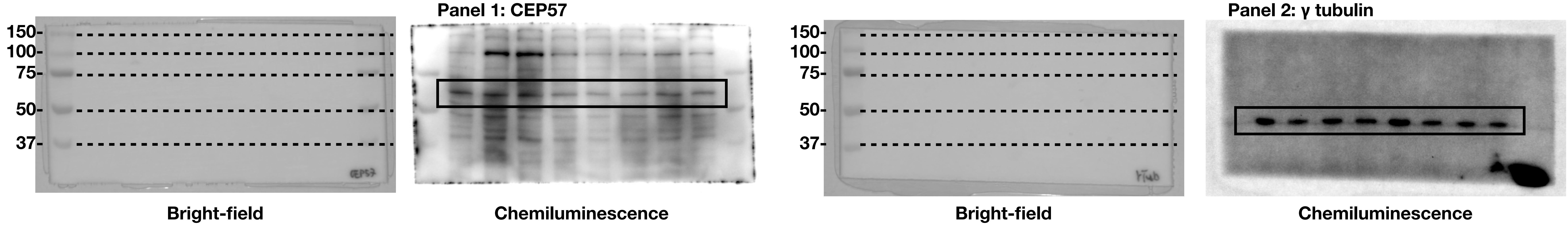

All blots were imaged using an Amersham Imager 600. Incremental exposure mode was used to capture chemiluminescence signals for the two blots. Bright-field images of the molecular weight ladder were acquired using manual mode with a 0.1 s exposure time.

Figure 7I

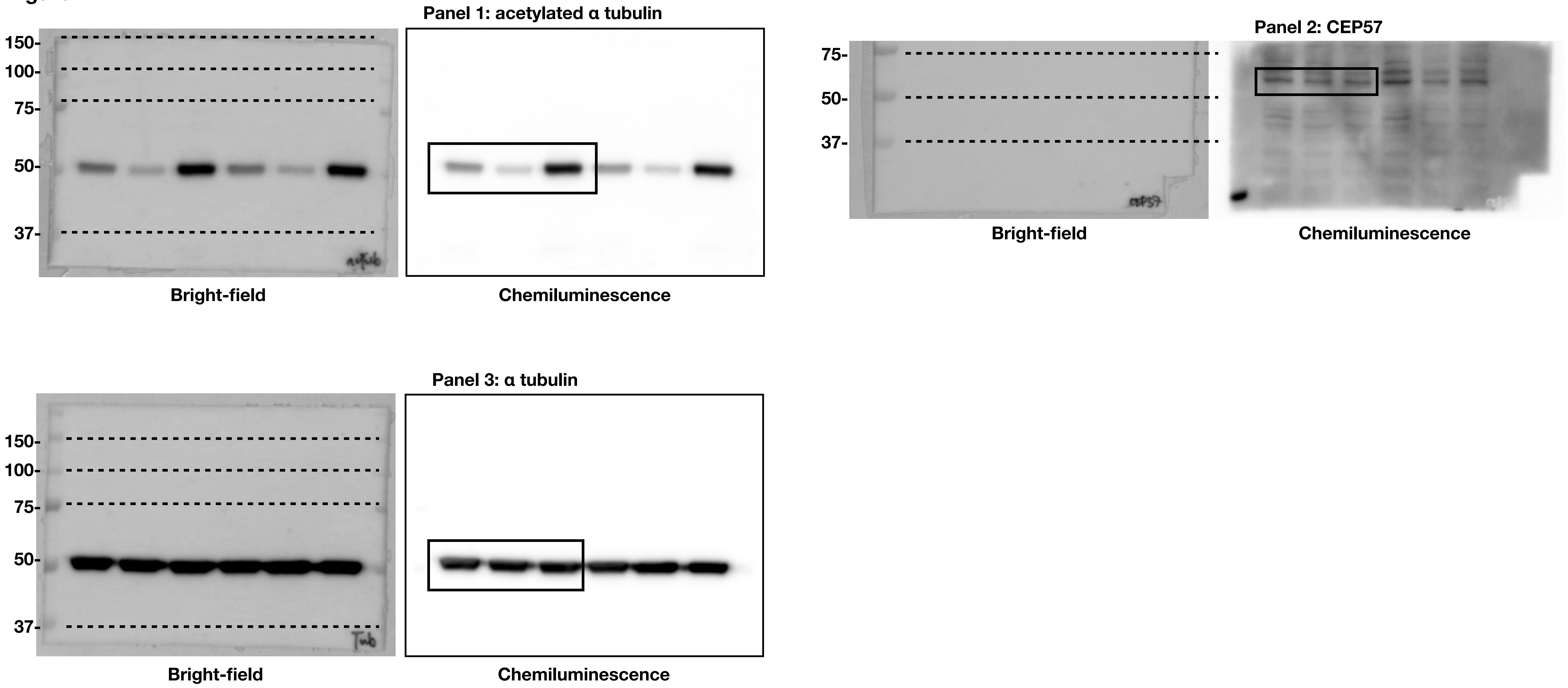

All blots were imaged using an Amersham Imager 600. 2 replicates were included in these 3 blots. For blots shown in panels 1 and 3, auto mode was used for image acquisition, and the overlay of the molecular weight ladder and chemiluminescence signals was automatically generated by the imager. For the blot shown in panel 2, incremental exposure mode was used to capture chemiluminescence signals. Bright-field images of the molecular weight ladder were acquired in manual mode with a 0.1 s exposure time.

Figure S1

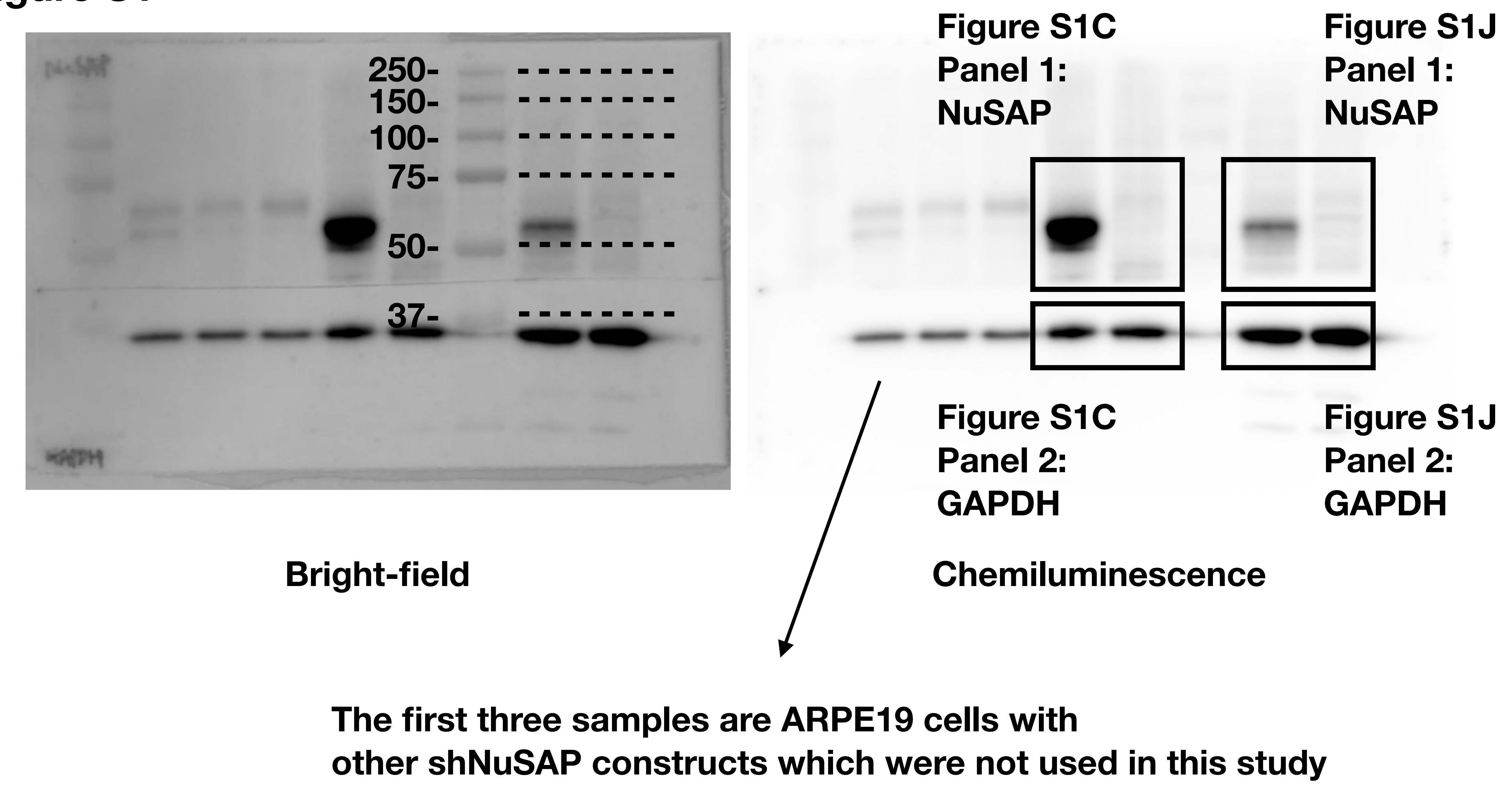

Figure S2C

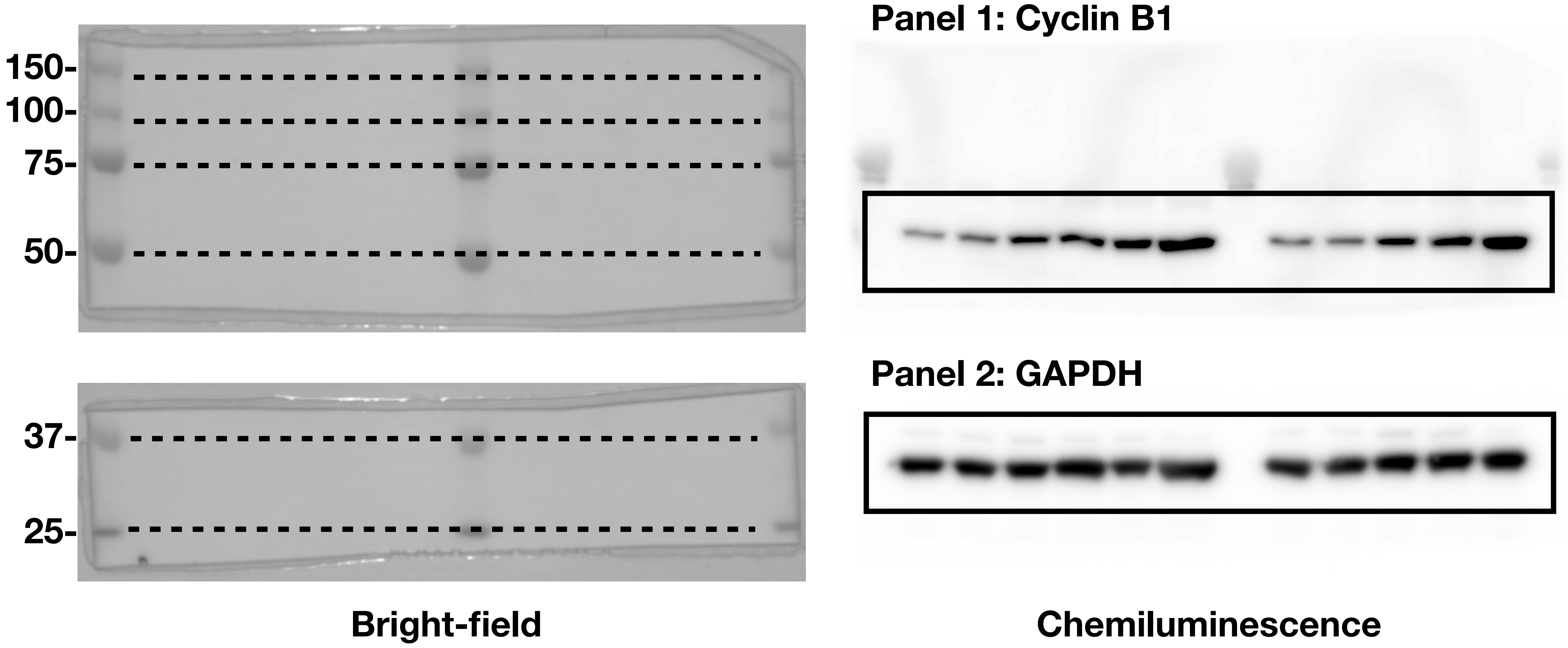

All blots were imaged using an Amersham Imager 600. For blot shown in Figure S1C and J, auto mode was used for image acquisition, and the overlay of the molecular weight ladder and chemiluminescence signals was automatically generated by the imager. For the blot shown in Figure S2C, incremental exposure mode was used to capture chemiluminescence signals. Bright-field images of the molecular weight ladder were acquired in manual mode with a 0.1 s exposure time.

Figure S5A

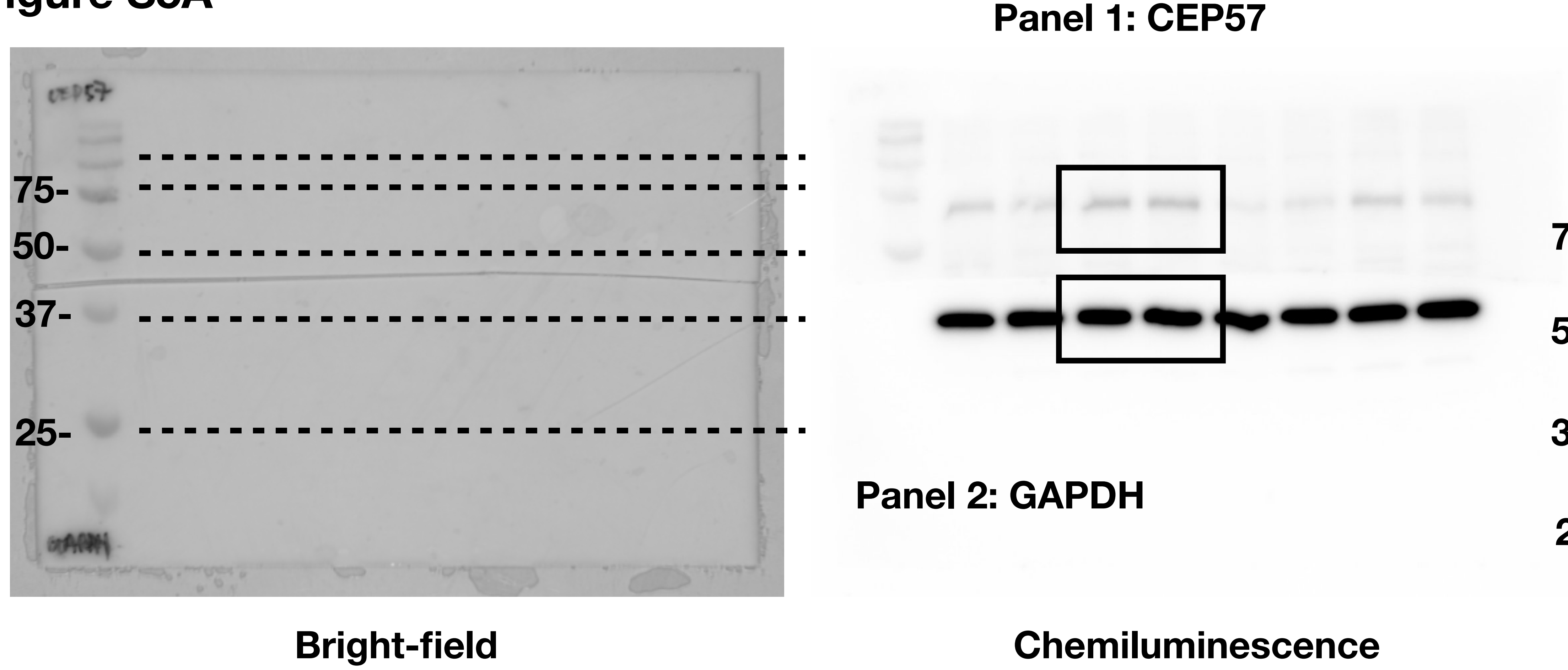

Four replicates were included in this blot for quantification.
A representative image (the second pair) is shown in the main text.

Figure S5B

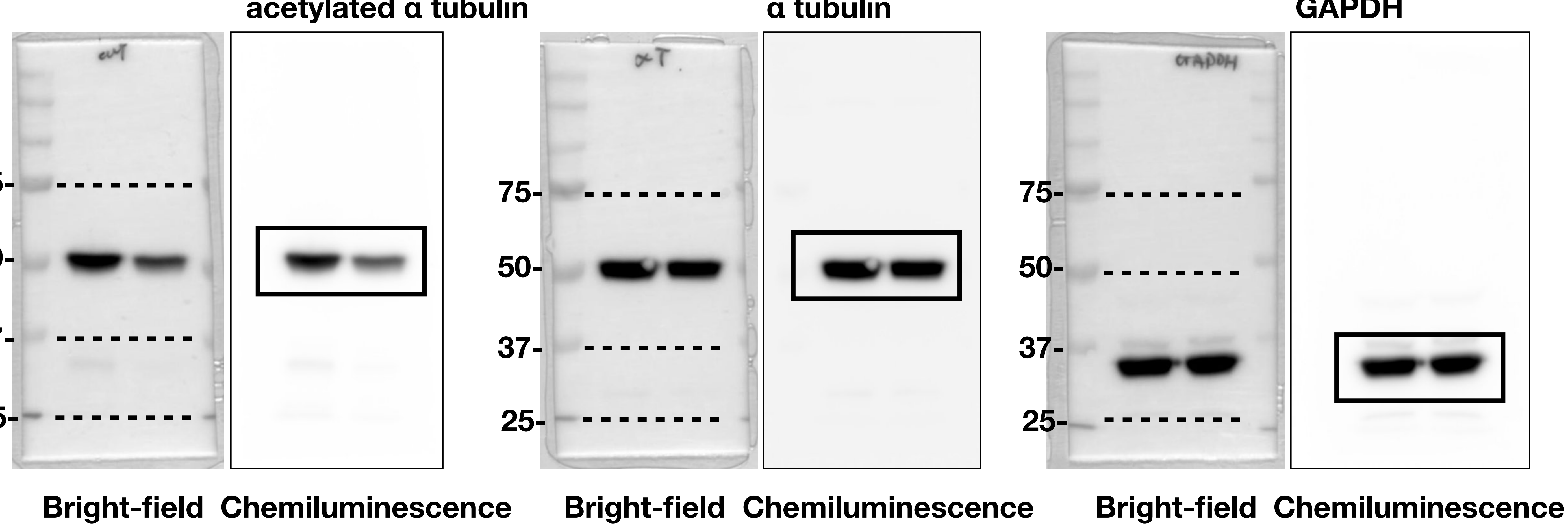

All blots were imaged using an Amersham Imager 600. For blots shown in Figure S5B, auto mode was used for image acquisition, and the overlay of the molecular weight ladder and chemiluminescence signals was automatically generated by the imager. For the blot shown in Figure S5A, incremental exposure mode was used to capture chemiluminescence signals. Bright-field images of the molecular weight ladder were acquired in manual mode with a 0.1 s exposure time.
